# Interventions to Address Moral Distress in Nurses and Other Healthcare Professionals: A Scoping Review and Evidence Gap Map

**DOI:** 10.3390/healthcare14183023

**Published:** 2026-09-15

**Authors:** Kordula Lang-Illievich, Thomas Danninger, Sascha Hammer, Johanna Lang, Istvan S. Szilagyi, Helmar Bornemann-Cimenti

**Affiliations:** 1 Department of Anesthesia and Intensive Care Medicine, Klinikum Güssing, 7540 Güssing, Austria; kordula.lang-illievich@medunigraz.at; 2 Department of Anesthesiology and Intensive Care Medicine, Medical University of Graz, 8010 Graz, Austria; sascha.hammer@medunigraz.at; 3 Department of Anesthesiology, Perioperative Medicine and General Intensive Care Medicine, Paracelsus Medical University, 5020 Salzburg, Austria; t.danninger@salk.at (T.D.);; 4 Department of Psychiatry, Psychosomatics and Psychotherapeutic Medicine, Medical University of Graz, 8010 Graz, Austria; istvan.szilagyi@medunigraz.at

**Keywords:** moral distress, nursing, scoping review, evidence gap map, ethics consultation, intervention

## Abstract

**Highlights:**

**What are the main findings?**

- Interventions, intended to influence the moral-distress cluster on education and on ethics deliberation or consultation, are delivered predominantly to individuals and teams, and act around the moral event or downstream of it.
- Only one of 32 studies intervened preventively at the source of moral distress, and only five operated at the organisation or system level.

**What are the implications of the main findings?**

- The empty regions of the evidence gap map identify where interventions have not yet been evaluated, rather than where they have been shown to fail.
- Future evaluations should be directed at the source of moral distress and at the organisational level, and should adopt validated measures and designs capable of controlling confounding.

**Abstract:**

**Background/Objectives**: To map interventions intended to influence moral distress in nurses and other healthcare professionals by intervention modality, organisational level, and point of action in the moral-distress process, and to appraise the methodological quality of the underlying studies. **Methods**: Scoping review with an evidence gap map, reported in accordance with the PRISMA Extension for Scoping Reviews. PubMed, Ovid (MEDLINE, Embase, Cochrane CENTRAL and the Cochrane Database of Systematic Reviews), CINAHL and the Web of Science Core Collection were searched without language, date or publication-status restrictions (databases searched to 13 July 2026), supplemented by forward and backward citation searching. Records were screened, charted and coded independently by two reviewers (coding kappa 0.75 to 0.84 by dimension). Charting used best-fit framework synthesis, and methodological quality was appraised with the Mixed Methods Appraisal Tool 2018 at the item level. Findings were synthesised descriptively through frequency counts and cross-tabulation and displayed as an evidence gap map. **Results**: Thirty-two studies were included, most from the United States (20 of 32). Interventions clustered on education (13 of 32) and ethics deliberation or consultation (9 of 32), were delivered predominantly at the individual or team level (27 of 32), and acted around the moral event (24 of 32) or downstream of it (7 of 32); only one intervened at the source. A direct moral-distress measure was used in 24 of 32 studies, and the fourth, design-specific appraisal criterion was clearly met in only 2 of 32. **Conclusions**: The field acts around and after the moral event but rarely at its source or at the organisational level. Source-directed and organisational interventions and validated measurement, are the priorities for future research.

## 1. Introduction

Moral distress was first delineated by Jameton [1]. It arises when a clinician knows which course of action is ethically right, but is prevented from taking it by institutional or structural constraint. The conditions that give rise to moral distress therefore lie in the environment in which care is delivered, and not in the moral character of the individual clinician. The distress itself, however, is an individual response: it is the clinician who perceives the constraint as morally significant and who carries its consequences. Keeping the antecedent conditions and the individual experience apart matters for the design of interventions, because the two invite different remedies.

Moral distress is both common and consequential. In an institution-wide survey of 592 clinicians, it was present in every professional group, and was most pronounced among those engaged in direct patient care [2]. A systematic review of its correlates found it to be associated with a poor ethical climate and limited collaboration, with low work satisfaction and engagement, and with low psychological empowerment and autonomy, and concluded that it bears on both the wellbeing of clinicians and their retention in post [3]. Among critical care providers, moral distress has been shown to be independently associated with severe burnout [4]. Sustained moral distress is further reported to erode the wellbeing of nurses, to be associated with the intention to leave high-acuity areas and the profession, and to bear on the quality of patient care [5,6]. Against a background of persistent workforce shortage, even a modest contribution to attrition carries organisational weight, and the mitigation of moral distress has accordingly become an objective of clinical governance and not merely a subject of research.

Over the past few years, several syntheses have mapped the intervention literature. They converge on a consistent picture: educational interventions predominate, the methodological quality of the primary studies is limited, and no single intervention can yet be regarded as demonstrably efficacious [5,6,7,8,9,10,11]. Two of these reviews concentrated on the intensive care setting [6,7], as have subsequent reviews in adult and paediatric intensive care [10,11], while a further review took a broader hospital-wide view and drew particular attention to the scarcity of action at the organisational level [8]. A scoping review of recommendations and strategies for nurses across care settings likewise identified no consistent pattern of effective strategies [9]. The cumulative message is that interventions exist and are being studied, but that the evidence supporting them remains fragile and heterogeneous.

What these syntheses share is a taxonomy organised by intervention type and an appraisal oriented towards effectiveness. None of them asks at which point in the moral-distress process an intervention acts. Morley, Bradbury-Jones and Ives proposed an empirically grounded model of that process, developed expressly to inform the design of interventions [12]; to our knowledge, however, no synthesis has used it to ask where along the trajectory the field actually acts, nor has any cross-tabulated the point of action against intervention modality.

The distinction is not merely descriptive. An intervention that removes a constraint before it produces distress addresses the phenomenon at its origin and is, by its nature, a matter of policy, authority or staffing, and therefore of organisational responsibility. An intervention that strengthens perception, ethical competence and agency around the moral event leaves the constraint in place, but changes what the clinician is able to do with it. An intervention that addresses the residue afterwards accepts both, and mitigates their consequences. Two bodies of evidence that appear identical when catalogued by intervention type may therefore differ fundamentally in what they can be expected to achieve, and a field that acts predominantly after the event will accumulate evidence that looks abundant while leaving the causes of moral distress untouched. Locating interventions along the process renders that structural feature of the evidence visible, and identifies the regions in which intervention development and evaluation are most needed.

This scoping review addresses that gap. Its aim is to map interventions intended to influence moral distress in nurses and other healthcare professionals across three dimensions, namely intervention modality, organisational level, and point of action in the moral-distress process; to display the result as an evidence gap map; and to appraise the methodological quality of the underlying studies. The purpose is descriptive rather than evaluative, namely, to show where the field concentrates its efforts and where it does not, so that future work can be directed towards the emptiest regions of the map.

## 2. Materials and Methods

This scoping review followed the methodological framework of Arksey and O’Malley [13], as refined by Levac et al. [14]. It is reported in accordance with the PRISMA Extension for Scoping Reviews (PRISMA-ScR) [15]; the completed checklist is provided as Supplementary File S2. A scoping design was chosen deliberately. The objective was to map how interventions are distributed across the moral-distress process, not to estimate their effectiveness, which the heterogeneity and the methodological limitations of the primary literature would not support. The protocol was registered with the Open Science Framework (DOI 10.17605/OSF.IO/AKCZG).

### 2.1. Search Methods

The search was constructed as a two-block strategy that combined the phenomenon of interest, moral distress, together with its grammatical variants and the cognate terms moral residue, moral suffering and moral injury, with the population of health professionals. No intervention block was used. Intervention terminology in this field is heterogeneous and frequently absent from titles and abstracts, so an intervention facet would have cost recall disproportionately in a small literature; eligibility with respect to the intervention was therefore established at screening rather than in the search. The professional block deliberately spanned the full range of health professions as well as generic staff, provider and care-team designators, consistent with the broad population criterion, and no restriction by professional group was applied at screening. Free-text terms were searched in title and abstract fields without controlled vocabulary, because moral distress has no descriptor in MeSH, in Emtree or in the CINAHL Subject Headings.

Four platforms were searched: PubMed; an Ovid multi-file search covering MEDLINE, Embase, the Cochrane Central Register of Controlled Trials and the Cochrane Database of Systematic Reviews; CINAHL on the EBSCOhost platform; and the Web of Science Core Collection through its advanced search interface. All databases were searched from the earliest record available on each platform (MEDLINE from 1946 and Embase from 1974) to 13 July 2026, with a final update of all searches on 14 July 2026, without language, date or publication-status limits. The database searches were supplemented by forward and backward citation searching of the included studies and of the prior syntheses in July 2026. Searching is reported in accordance with PRISMA-S [16]; the strategies for all four platforms, reproduced line by line as executed, together with the platform-specific deviations from a strict title and abstract restriction, are provided as Supplementary File S1.

### 2.2. Inclusion and Exclusion Criteria

Eligibility was framed with the population, concept and context (PCC) framework recommended for scoping reviews, and each element was operationalised before screening.

Population: healthcare professionals of any discipline, whether in training or in practice, including nurses, physicians, midwives, pharmacists, chaplains and allied health professionals. Studies with mixed interprofessional samples were eligible and no restriction by professional group was applied, so that the predominance of nursing samples among the included studies reflects the primary literature rather than a selection decision.

Concept: any structured intervention with the declared or evident aim of influencing moral distress. Both elements of this criterion were operationalised. An intervention was counted as structured where it had a described and repeatable form that is an identifiable content, format, provider and mode of delivery; incidental support, informal collegial exchange and general features of the working environment that were not delivered as a defined intervention were not eligible. The aim of influencing moral distress counted as declared where the report named moral distress, or one of its cognate terms, as a target of the intervention in its stated aim, its rationale or its evaluation. In the absence of such a statement, the aim counted as evident only where the report both framed the intervention explicitly as a response to morally constrained practice and reported an outcome that the authors themselves interpreted in relation to moral distress. Studies addressing an adjacent construct alone, such as resilience, ethical competence or work climate, without any explicit link to moral distress, were not eligible; conversely, studies that named moral distress as the target but evaluated it by means of a surrogate construct remained eligible, and the outcome status of every included study is encoded in the evidence gap map (Supplementary File S3).

Context: any clinical care setting, including acute, critical, long-term and community care.

Study design: empirical intervention studies of qualitative, quantitative or mixed-methods design. Reviews, commentaries, conceptual papers, protocols without results, and descriptive studies of the prevalence or correlates of moral distress that evaluated no intervention were excluded. No language, date or publication-status restrictions were applied.

### 2.3. Quality Appraisal

Critical appraisal is not a routine component of scoping reviews, and is ordinarily undertaken only where the aim of the particular review requires it [17]. That condition is met here on two counts: the appraisal of methodological quality was a stated objective of the review in its own right, and the central claim of the map, namely that certain regions are under-studied rather than ineffective, is intelligible only if the robustness of the studies occupying the populated regions is made visible alongside their distribution. Methodological quality was appraised with the Mixed Methods Appraisal Tool (MMAT), version 2018 [18], which accommodates all five study designs represented in the corpus within a single instrument. Consistent with the developers’ guidance, no overall numerical score was computed, and the item-level pattern of ratings was retained. The appraisal describes the corpus: it did not affect eligibility, and studies were neither ranked, weighted nor filtered on quality grounds.

### 2.4. Data Charting

Data were charted on a single form, developed a priori, piloted on the first five studies and frozen before full application. For each study, the form captured bibliographic and contextual details (authors, year, country, care setting), the sample (professional groups and size), the study design and its MMAT category, a structured description of the intervention aligned with the TIDieR checklist [19] (components and content, provider and facilitation profile, format, dose and duration, trigger, embedding in routine work, and any tailoring), the outcome construct and the instrument used, the reported direction of effect, and the theoretical framework named by the authors. The charting form and the complete charted dataset for all 32 studies are provided as Supplementary File S4. Charting was followed by the coding of the three mapping dimensions; the two steps are reported separately below in order to keep data extraction and classification distinct.

### 2.5. Coding of the Mapping Dimensions

Coding followed a best-fit framework synthesis [20]: a category system derived from convergent prior syntheses [5,6,7,8] was applied deductively and extended inductively where studies did not fit. Each study was coded on three dimensions, abbreviated throughout the text and figures as M (intervention modality; M1–M7), L (intervention level; L1–L3) and A (point of action in the moral-distress process; A1–A3). Dimension one, intervention modality, comprised seven mutually exclusive primary categories (M1 education; M2 facilitated case discussion and debriefing; M3 ethics deliberation and consultation services; M4 interprofessional rounds; M5 reflective and narrative methods; M6 resilience and mindfulness methods; M7 structural-organisational measures), with an optional secondary modality. Dimension two, intervention level, distinguished the individual (L1), the team or unit (L2) and the organisation or system (L3); it was coded at the level at which the intervention was delivered and structurally anchored, not at the level at which the cause of moral distress was located, and it is conceptually anchored in Jameton’s account of moral distress as arising from institutional constraint [1]. Dimension three, point of action in the moral-distress process, was operationalised from the model of Morley et al. [12]: A1, preventive and at the source; A2, supportive-enabling around the moral event; and A3, processing-downstream, addressing the reactive residue, with a mandatory primary and an optional secondary code.

A dominance rule governed the assignment of the primary modality: the temporally and substantively predominant component was coded as primary; where components were inseparable, an integrated-programme flag was set and the primary modality was assigned by the largest session or time share and, at genuine parity, by the authors’ stated principal aim. Cross-cutting tags, aligned with the TIDieR checklist [19], captured empowerment as an explicit aim, facilitation profile, dose, trigger, embedding, outcome construct and theoretical framework. Empowerment was deliberately coded as a cross-cutting tag rather than as a modality, because it is realised through several intervention forms; for the same reason, it was not elevated to a fourth mapping dimension, the scoping objective being to locate interventions rather than to model their mechanisms. The full coding framework, comprising the operational definition of each category, its delimitation against neighbouring categories, anchor examples, the dominance rule, the cross-cutting tags and the standing adjudication log of borderline decisions, is provided as Supplementary File S3.

### 2.6. Reliability and Software

Screening, data charting and coding were carried out as three separate steps, each in duplicate. All records were screened independently by two reviewers at both stages, and overall inter-screener agreement was 94%. Data charting, that is the extraction of study and intervention characteristics on the frozen form, was likewise performed independently by two reviewers, and the two completed forms were reconciled field by field against the full text. The coding of the three mapping dimensions was then performed independently by two reviewers on the basis of the reconciled charting form, and inter-rater reliability was computed separately for each dimension: Cohen’s κ was 0.75 for intervention modality (M), 0.84 for intervention level (L) and 0.79 for point of action (A), corresponding to substantial-to-almost-perfect agreement. Disagreements at every step were resolved by consensus, with a third reviewer available for any residual discrepancy. Records were managed and deduplicated in Zotero (version 9.0.6, Corporation for Digital Scholarship, Vienna, VA, USA); screening, data charting and coding were carried out on a structured Microsoft Excel workbook, which is reproduced as Supplementary File S4; Cohen’s κ was computed in R (version 4.6.1, R Foundation for Statistical Computing, Vienna, Austria); and the evidence gap map and the appraisal plot were produced with Matplotlib (version 3.11.2) in Python.

### 2.7. Synthesis

Each study contributed one primary modality, one intervention level and one primary point of action, so that the charted material formed a study-level dataset over three categorical dimensions. The synthesis proceeded in three steps. Marginal frequencies were first computed for each dimension separately. The two cross-tabulations that carry the argument of the review, modality against level and modality against point of action, were then constructed. Finally, the three dimensions were displayed jointly as an evidence gap map of modality against point of action, stratified by intervention level, so that the type of intervention, the level at which it was delivered and the point at which it acts can be read simultaneously. In the map, bubble area is proportional to the number of studies; the surrounding ring encodes the share of validated as against surrogate or non-validated moral-distress measurement; and a gold ring marks empowerment as an explicit intervention aim. The map displays primary codes only, so that each study appears in exactly one cell. A single primary classification was preferred to multiple coding for three reasons. First, the question of the review concerns the distribution of studies, so that the cell counts must sum to the corpus and the bubble areas must retain a fixed denominator, which multiple coding would forfeit. Second, secondary codes vary in weight, from an integrated second component to a subsidiary aim mentioned in a single sentence, and counting them on a par with primary codes would over-represent peripheral components in precisely the sparse regions that the map is designed to expose. Third, the dominance rule and the adjudication log make the primary assignment reproducible and contestable, whereas the threshold for recording a secondary code is necessarily looser. Secondary modality and secondary point-of-action codes were nevertheless retained in the charted dataset and are used narratively, and the dependence of the principal pattern on this choice was examined in a sensitivity analysis, in which the marginal frequencies and the cross-tabulation of modality against point of action were recomputed under multiple coding, each study being counted in every category for which it held a primary or a secondary code (Supplementary File S5). In addition, because the fourth MMAT criterion is design-specific and assesses the control of confounding only in the non-randomised category, the reported direction of effect was tabulated against study design, distinguishing randomised trials, non-randomised studies with a comparison condition and uncontrolled designs, rather than against that criterion (Supplementary File S6). A narrative synthesis accompanied the map.

## 3. Results

### 3.1. Study Selection

**Figure 1:**
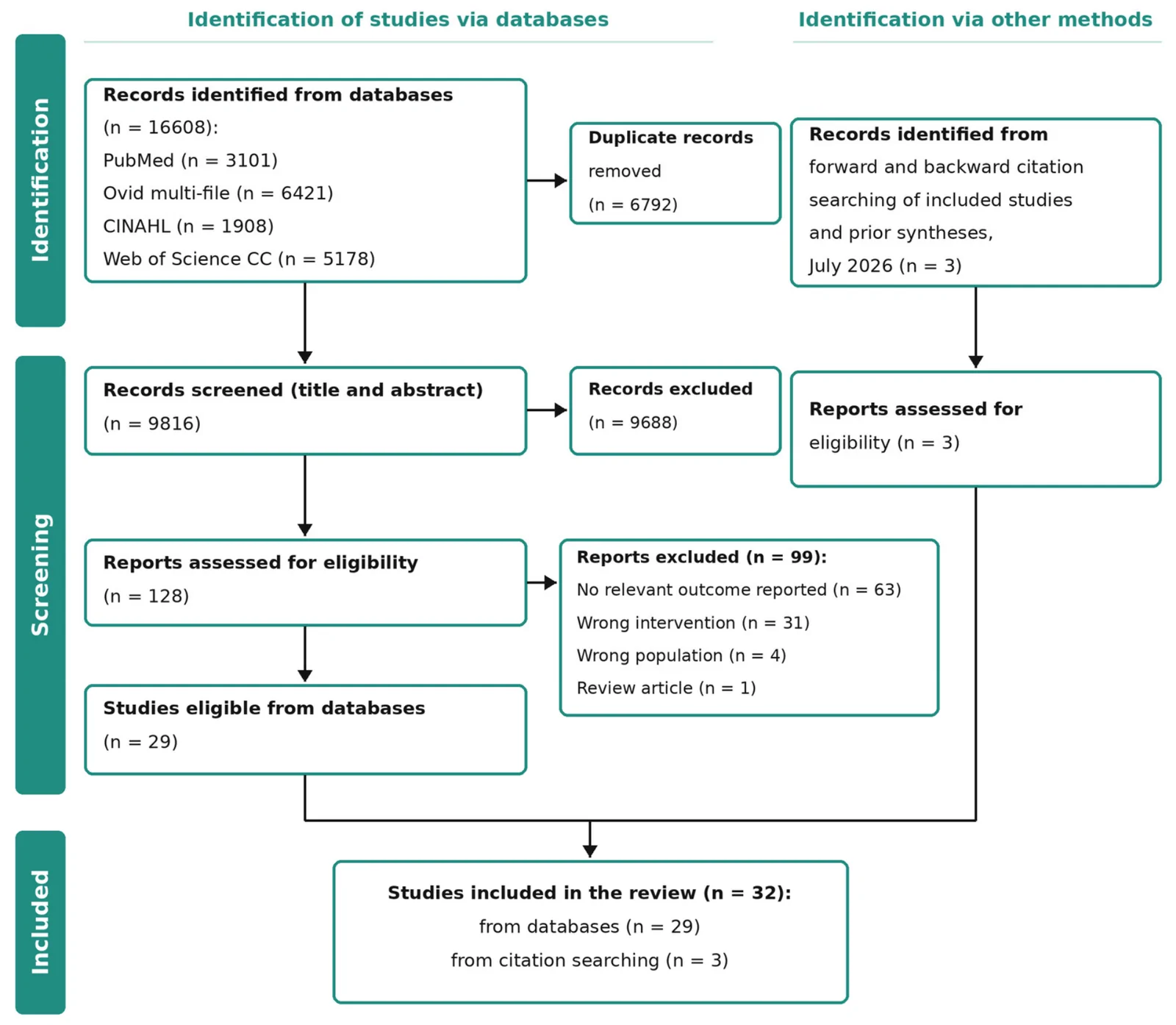
PRISMA-ScR flow diagram of study identification and selection. CC, Core Collection; Ovid multi-file, MEDLINE (including Epub Ahead of Print and In-Process records), Embase, Cochrane CENTRAL and the Cochrane Database of Systematic Reviews.

The searches returned 16,608 records (PubMed 3101; Ovid multi-file 6421; CINAHL 1908; Web of Science Core Collection 5178). After the removal of 6792 duplicates, 9816 unique records were screened on title and abstract, of which 128 were retained for full-text assessment; 29 met the eligibility criteria. The supplementary forward and backward citation search of the included studies and of the prior syntheses identified three further eligible reports, giving 32 included studies [21,22,23,24,25,26,27,28,29,30,31,32,33,34,35,36,37,38,39,40,41,42,43,44,45,46,47,48,49,50,51,52]; the three reports identified through citation searching were those of Brännström et al. [27], Tavakol et al. [47] and Wocial et al. [51]. The selection process is summarised in the PRISMA-ScR flow diagram (Figure 1).

### 3.2. Included Studies

Thirty-two intervention studies were included (Table 1) [21,22,23,24,25,26,27,28,29,30,31,32,33,34,35,36,37,38,39,40,41,42,43,44,45,46,47,48,49,50,51,52]. The evidence base was geographically concentrated: 20 of 32 studies originated in the United States, with the remainder from Iran (*n* = 4), Norway (*n* = 2), Canada (*n* = 2), and Sweden (*n* = 2), and one each from Switzerland and the Netherlands. Although the eligibility criteria admitted all healthcare professionals, the samples were dominated by nursing: in most studies, nurses constituted all or the clear majority of participants. By MMAT category, the corpus comprised 13 quantitative non-randomised studies, 10 mixed-methods studies, 4 randomised controlled trials, 3 qualitative studies, and 2 quantitative descriptive studies. Interventions were most often delivered as add-on activities and, less frequently, integrated into routine workflow; seven programmes combined inseparable components and were flagged as integrated [26,29,31,47,48,51,52].

### 3.3. Intervention Modality

**Figure 2:**
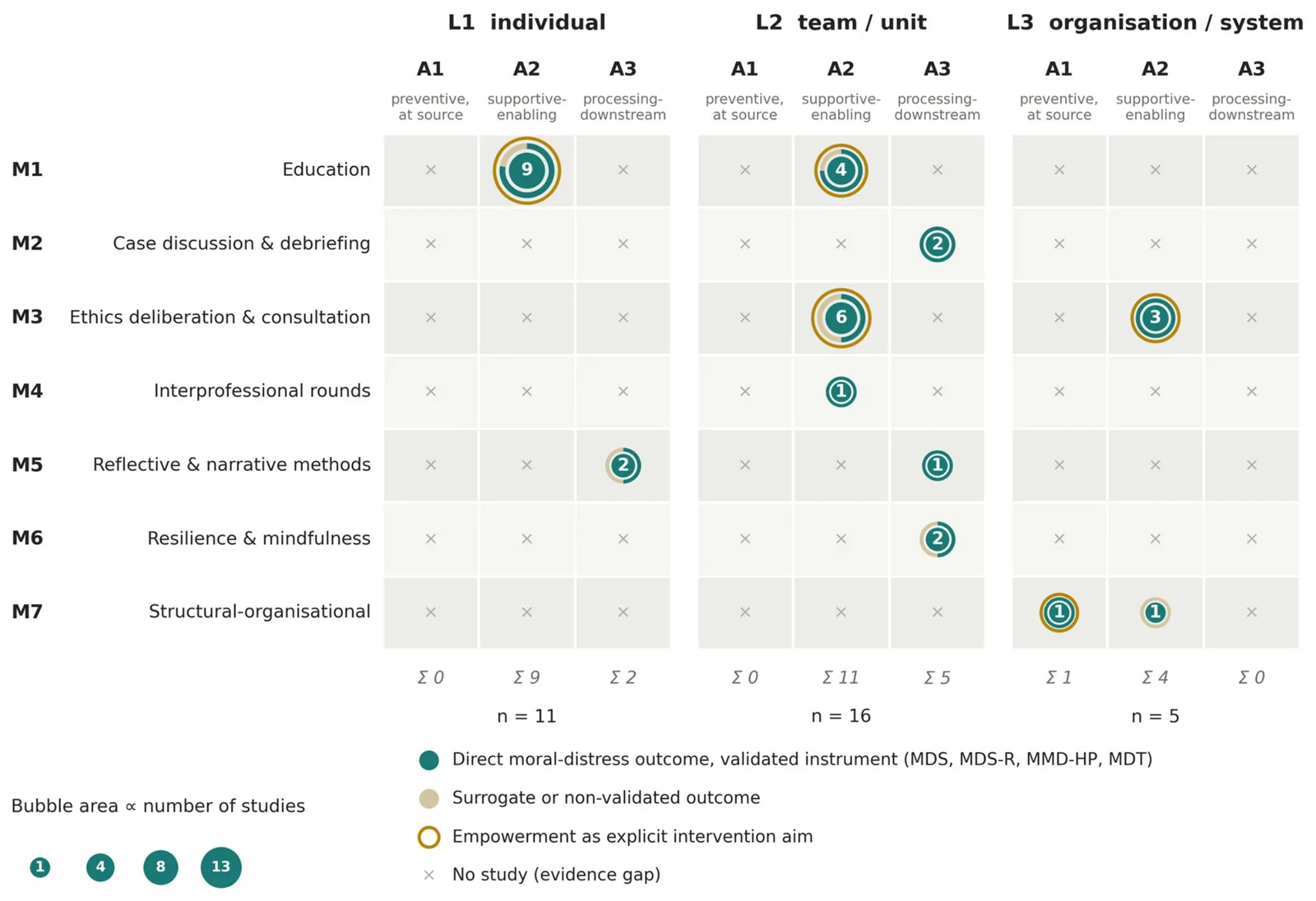
Evidence gap map of the 32 included studies: intervention modality (M1–M7) against point of action in the moral-distress process (A1 preventive, at the source; A2 supportive-enabling, around the moral event; A3 processing-downstream), stratified by intervention level (L1 individual; L2 team or unit; L3 organisation or system); each study is counted once, by its primary codes. Bubble area is proportional to the number of studies; the ring shows the share of validated (teal) versus surrogate or non-validated (sand) moral-distress measurement; a gold ring marks empowerment as an explicit intervention aim; a cross marks an empty cell.

Education (M1) was the dominant modality, accounting for 13 of 32 studies, followed by ethics deliberation and consultation services (M3, *n* = 9). As shown in Figure 2, the remaining modalities were sparsely populated: reflective and narrative methods (M5, *n* = 3), facilitated case discussion and debriefing (M2, *n* = 2), resilience and mindfulness methods (M6, *n* = 2), structural-organisational measures (M7, *n* = 2), and interprofessional rounds (M4, *n* = 1).

### 3.4. Intervention Level

Interventions were delivered predominantly at the individual (L1, *n* = 11) and the team or unit level (L2, *n* = 16); only five were established at the organisation or system level (L3). The joint distribution of modality, level and point of action is displayed in Figure 2: educational interventions were overwhelmingly individual (9 of 13), and the only organisation-level interventions were three of the nine ethics-consultation services and the two structural-organisational measures.

### 3.5. Point of Action in the Moral-Distress Process

The distribution across the moral-distress process was markedly uneven (Figure 2). Twenty-four of 32 interventions acted in a supportive-enabling capacity around the moral event (A2), and seven addressed the reactive residue downstream (A3). A single study intervened preventively at the source (A1): a policy change permitting nurses to initiate palliative-care consultation independently [25]. The downstream cluster (A3) comprised reflective and narrative methods (*n* = 3), facilitated debriefing (*n* = 2), and resilience or mindfulness bundles (*n* = 2). The largest single cell of the evidence gap map was educational interventions acting around the event (M1 × A2, *n* = 13), whereas the preventive column was almost entirely empty.

Under multiple coding (Supplementary File S5), the 32 studies contributed 70 study-cell entries. Action at the source (A1) rose from one to four studies, the three additional entries being secondary aims of two ethics-consultation services and one educational programme [32,34,47]; processing-downstream action (A3) rose from seven to twelve; and ethics deliberation or consultation (M3) rose from nine to thirteen, equal to education, whose count was unchanged. The distribution by intervention level, which is coded once per study, was unaffected. The preventive column thus remained the least populated region of the map under either coding strategy, and the pattern reported above did not depend on the choice of primary coding.

### 3.6. Empowerment and Outcome Measurement

Strengthening moral agency was an explicit aim in seven studies [21,24,25,32,34,47,51]. A direct moral-distress measure was used in 24 of 32 studies; of these, 23 employed a validated instrument (the Moral-Distress Scale, its revised version, the Measure of Moral-Distress for Healthcare Professionals (MMD-HP), or the Moral Distress Thermometer) and one an author-developed questionnaire [23]. The remaining eight relied on surrogate constructs, such as resilience, comfort, or psychosocial work climate.

### 3.7. Methodological Quality

**Figure 3:**
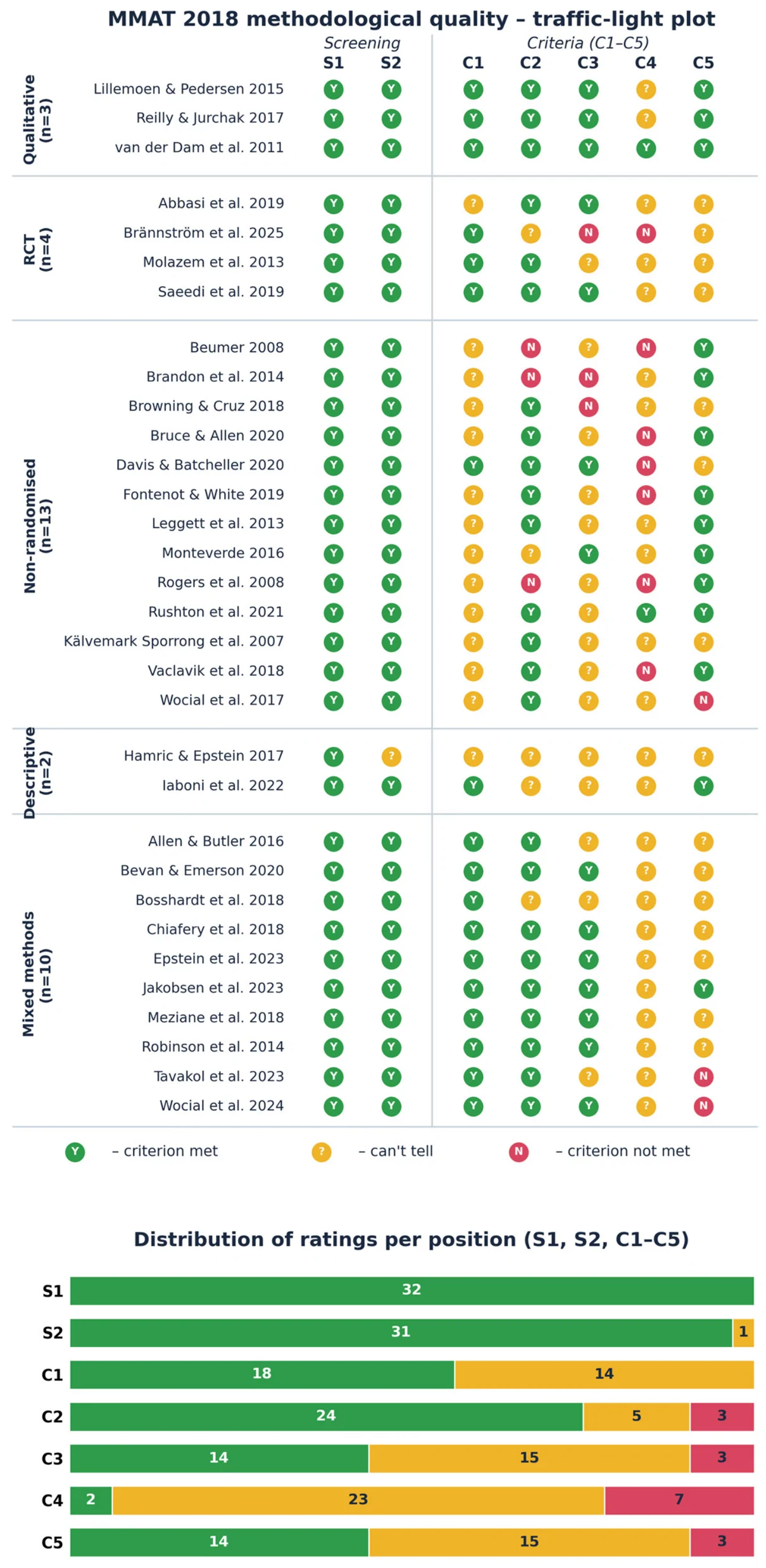
Methodological quality of the 32 included studies appraised with the Mixed Methods Appraisal Tool (MMAT), version 2018, shown as a traffic-light plot by study and criterion (S1–S2 screening; C1–C5 category-specific), with the distribution of ratings per position beneath. The fourth criterion is design-specific: it denotes the substantiation of the interpretation by the data in qualitative studies, the blinding of outcome assessors in randomised trials, the control of confounding in non-randomised studies, a low risk of non-response bias in descriptive studies, and the handling of divergences between quantitative and qualitative findings in mixed-methods studies. Studies are identified by first author and year. Qualitative studies (*n* = 3): Lillemoen and Pedersen 2015 [38], Reilly and Jurchak 2017 [42], van der Dam et al. 2011 [49]. Randomised controlled trials (*n* = 4): Abbasi et al. 2019 [21], Brännström et al. 2025 [27], Molazem et al. 2013 [40], Saeedi et al. 2019 [46]. Non-randomised studies (*n* = 13): Beumer 2008 [23], Brandon et al. 2014 [26], Browning and Cruz 2018 [28], Bruce and Allen 2020 [29], Davis and Batcheller 2020 [31], Fontenot and White 2019 [33], Leggett et al. 2013 [37], Monteverde 2016 [41], Rogers et al. 2008 [44], Rushton et al. 2021 [45], Kälvemark Sporrong et al. 2007 [52], Vaclavik et al. 2018 [48], Wocial et al. 2017 [50]. Quantitative descriptive studies (*n* = 2): Hamric and Epstein 2017 [34], Iaboni et al. 2022 [35]. Mixed-methods studies (*n* = 10): Allen and Butler 2016 [22], Bevan and Emerson 2020 [24], Bosshardt et al. 2018 [25], Chiafery et al. 2018 [30], Epstein et al. 2023 [32], Jakobsen et al. 2023 [36], Meziane et al. 2018 [39], Robinson et al. 2014 [43], Tavakol et al. 2023 [47], Wocial et al. 2024 [51].

Screening criteria were met almost universally: all 32 studies posed clear research questions (S1), and 31 provided data capable of addressing them (S2). Among the category-specific criteria, appropriateness of measurement together with baseline comparability (C2) was the most frequently satisfied, being met in 24 studies, whereas the fourth criterion was the consistent weak point. Across designs, this criterion captures the blinding of outcome assessors, control of confounding, risk of non-response bias, the handling of divergences between quantitative and qualitative findings, and the substantiation of the interpretation by the data; it was clearly met in only 2 studies, not met in 7, and indeterminable in 23. Because the criterion differs by design, this count aggregates five distinct items; it describes the item-level pattern of the corpus and is not a single index of confounding control, which the fourth criterion assesses only in the non-randomised category, where one of 13 studies met it. Of the 224 appraised item ratings, 135 were rated “yes”, 73 “can’t tell” and 16 “no”, so that incomplete reporting rather than documented methodological failure accounted for most of the uncertainty. The full item-level pattern is shown in Figure 3.

### 3.8. Reported Direction of Effect

Because a scoping review does not assess effectiveness, the direction of the reported effect was charted descriptively, as a characteristic of the corpus rather than as a synthesis. Among the 21 studies that used a direct moral-distress measure and permitted a usable inference, eight reported a statistically significant reduction, four a mixed pattern across measures or subscales, eight no significant change and one an increase; the remaining studies either measured moral distress without being able to evaluate it or relied on surrogate or qualitative outcomes. Reductions were confined to educational and ethics-consultation interventions and a single mindfulness bundle. By study design, a reduction was reported by two of the four randomised trials [21,40], by one of the five non-randomised studies with a comparison condition [32], and by five of the twelve uncontrolled designs [30,41,43,47,48] (Supplementary File S6). The study-level detail is reported in Supplementary File S4.

## 4. Discussion

### 4.1. Principal Findings

When the 32 included interventions were located along the moral-distress process rather than merely catalogued by type, the activity of the field proved strikingly uneven. Interventions concentrated on education and on ethics deliberation or consultation were delivered predominantly to individuals and teams and acted almost entirely around the moral event or downstream of it. Only one study intervened at the source of moral distress before it arose, and only five operated at the organisation or system level. A validated moral-distress instrument underpinned the outcome measurement in 23 of the 32 studies, while the remainder relied on surrogate constructs or on non-validated measures. This distribution, rather than the effectiveness of any single intervention, is the principal finding of the review.

Because the map is built on the primary code assigned to each study, this distribution is contingent on the framework by which those codes were assigned. The coding framework is therefore reported in full, with its operational definitions, its delimitation criteria and its adjudication log, as in Supplementary File S3, so that the classification underlying the central finding can be inspected and, where necessary, contested. The sensitivity analysis under multiple coding (Supplementary File S5) indicates that the principal pattern does not depend on this choice: even when every secondary code is counted, action at the source rises only from one to four studies, and the organisational level, which is coded once per study, is unaffected.

The near-absence of source-directed interventions warrants a cautious reading. Because the map records the primary point of action, it necessarily understates preventive activity that is present only as a secondary aim; two ethics-consultation services, for instance, addressed causal barriers alongside their supportive function. Similarly, one educational programme incorporated ward-level management meetings intended to correct workload and task-allocation processes at their source [47]. The preventive column is therefore sparse rather than truly empty, although even on a generous reading it remains the least populated region of the map.

A secondary and explicitly exploratory observation concerns the interventions that framed empowerment, understood as the strengthening of moral agency, as an explicit aim. Although only seven studies did so, they were disproportionately those that reached towards the least populated regions of the map: three operated at the organisation or system level, and four addressed, as a primary or secondary purpose, the source of moral distress, including the single study that intervened preventively at the source. The observation is best treated as hypothesis-generating rather than as support for the central argument of the review. The studies are few, the coding of an explicit aim is necessarily interpretive, and a substantial part of the pattern is attributable to a single moral-distress consultation programme and its evaluations. With those reservations, the pattern is at least consistent with Jameton’s account of moral distress as constrained agency, and it suggests a hypothesis worth testing: that researchers who set out to restore agency are more inclined to engage the structural and preventive levels at which constraint originates.

### 4.2. Relation to Previous Evidence

These findings sit comfortably alongside the recent syntheses of the intervention literature. Morley and colleagues, Imbulana and colleagues, Emami Zeydi and colleagues, and Amos and Epstein each concluded that educational interventions dominate, that the evidence base is methodologically fragile, and that no single intervention can yet be considered efficacious [5,6,7,8]. Subsequent reviews in adult and paediatric intensive care, and a scoping review of nurse-directed interventions across care settings, reached the same conclusion [9,10,11]; Chen and colleagues additionally distinguished individual from institutional interventions, a distinction that the level dimension of the present review extends and formalises [10]. The present review reproduces that descriptive picture but adds an analytic axis: by cross-tabulating modality against the point of action in the model of Morley, Bradbury-Jones and Ives [12], it renders visible a structural gap that a catalogue of intervention types conceals. Amos and Epstein observed that action at the organisational level is rare [8]; the counts reported here, five of 32 at the organisation or system level and one of 32 at the source, locate and quantify that scarcity.

A pattern in the reported directions deserves brief comment, offered as an exploratory observation about the corpus and not as an assessment of effectiveness, which this review does not undertake. Because the fourth MMAT criterion is design-specific and assesses the control of confounding only in non-randomised studies, the reported directions were examined by study design rather than by that criterion (Supplementary File S6). Among the 21 studies that used a direct moral-distress measure and permitted a usable inference, a reduction was reported by two of the four randomised trials [21,40], by one of the five non-randomised studies with a comparison condition [32], and by five of the twelve uncontrolled designs [30,41,43,47,48]. The remaining controlled studies reported no change, among them the only cluster-randomised trial in the corpus [27], the single non-randomised study in which confounding was demonstrably accounted for [45], and a two-phased ethics-conversation intervention evaluated between conditions [51]. Reductions are therefore not confined to weak designs. Most of them, however, derive from designs in which regression to the mean, secular trends and expectancy cannot be separated from the effect of the intervention, and in neither of the two randomised trials that reported a reduction could the blinding of outcome assessment be established from the report. The number of evaluable studies in each stratum is small, and the pattern is offered as a description of the corpus. The narrow conclusion is that the positive signal in this literature is thin and rests mostly on uncontrolled designs, and that claims of effectiveness should be treated with corresponding caution until they have been replicated under controlled conditions.

### 4.3. Why the Evidence Is Skewed

Several factors may explain why the evidence clusters where it does. The explanations that follow are theoretical: they are consistent with the distribution reported here, but they are not demonstrated by it, and the review can describe the pattern without being able to account for it. Interventions that act around or after the moral event are typically add-on activities, deliverable to individuals within a single unit and a single study period, and they align neatly with the pre-and-post designs and individual-level scales that the field has adopted. Interventions at the source, by contrast, require changes to authority, staffing or policy; they are slower to mount, harder to attribute, and less tractable to an instrument that measures individual experience. It is conceivable, on this reading, that the measurement tools quietly shape the interventions that are studied, since a scale calibrated to individual distress invites individual-level responses; the present data can neither support nor refute that conjecture. A more prosaic explanation is equally available, namely that researchers pursue what is feasible within the constraints of a funded study rather than what the theory of moral distress would prioritise.

### 4.4. Strengths and Limitations

The review has several strengths. The charting frame was specified a priori, piloted, and frozen before full application; its categories were anchored in the convergent terminology of prior syntheses through a best-fit framework synthesis [20]; and its borderline decisions were fixed in a standing adjudication log, which addresses the objection most often raised against such schemes. The framework and the log are published in full, so that every classification can be traced and contested. Aligning the cross-cutting tags with the TIDieR checklist [19] gave the intervention descriptions a reproducible basis, and appraising quality with the MMAT at the item level, without an inappropriate composite score, preserved the pattern of methodological strength and weakness.

Its limitations qualify the interpretation. Mapping each study by a single primary modality and primary point of action under-represents integrated and multi-component programmes, of which seven were identified, although the sensitivity analysis under multiple coding indicates that the principal pattern is not an artefact of this choice. The corpus is geographically concentrated, with 20 of 32 studies from the United States, which limits transferability. Much of the appraisal uncertainty reflected incomplete reporting rather than demonstrated methodological failure, so the quality statements are deliberately conservative. In addition, the eligibility criterion of a declared or evident aim of influencing moral distress will have excluded structural measures, such as changes to staffing or policy, that plausibly act at the source but are neither framed nor evaluated in terms of moral distress; the sparseness of the preventive column therefore partly reflects this selection, although the constraint is difficult to avoid, since interventions without a stated or measured link to the construct cannot be identified systematically. Finally, the search relied on free-text terms in title and abstract fields and deliberately omitted an intervention facet; this maximised recall for the phenomenon, but studies that evaluate an intervention relevant to moral distress without naming the construct in the title or abstract will not have been retrieved. One of the included trials, added through citation searching, carried no nursing or professional-group terminology in its title or abstract and was therefore structurally invisible to the two-block strategy despite naming the phenomenon [27]. Publication and reporting bias also deserve mention, although a scoping review can register but not formally assess them. The empty regions of the map record the absence of published evaluations, not necessarily the absence of attempts: organisational or source-directed initiatives that fail, or that are undertaken as quality improvement rather than research, are plausibly less likely to reach publication than circumscribed educational studies. The same asymmetry may operate on the reported directions, since favourable findings are more readily published; the predominance of uncontrolled designs among the studies reporting reductions may therefore partly reflect selective reporting rather than intervention effects. The scoping design precludes any inference about effectiveness or comparative effectiveness.

Taken together, these constraints delimit what the empty regions of the map can be taken to show. They describe the interventions that have been identified and evaluated in the published literature under the eligibility criteria and the coding framework of this review; they do not describe the interventions that exist in practice. Statements about the scarcity of source-directed and organisational interventions should be read in that restricted sense.

## 5. Conclusions

Locating interventions along the moral-distress process, rather than cataloguing them by type, reveals a field whose energies are unevenly deployed. Effort concentrates on education and on ethics deliberation or consultation is directed at individuals and teams, and gathers around the moral event or in its aftermath. The source of moral distress and the organisational or system level remain very largely unrepresented among published evaluations. Outcome measurement is heterogeneous: most studies used a validated instrument, but others relied on surrogate constructs. This distribution, and not the demonstrated efficacy of any single modality, is the substantive result of the review.

The contribution is accordingly twofold and deliberately modest. Conceptually, the review shows what becomes visible when interventions are located in the process that generates moral distress rather than sorted by type, namely a structural asymmetry between action at the source and action after the event that a taxonomy of intervention types conceals. Practically, it identifies the regions in which interventions have not yet been evaluated, and which therefore merit development and evaluation first. Because the design is a scoping review, neither contribution extends to any claim about effectiveness: the sparsely populated regions of the map record an absence of published evaluations rather than an established want of effect, and the reductions in moral distress that several studies report derive mostly from uncontrolled designs, so that the positive signal in this literature must be read with corresponding reserve.

The practical consequence is a research agenda defined by the empty rather than the crowded cells of the map. Priority belongs to interventions that act preventively at the source of moral distress and at the organisational or system level, for it is precisely there that the account of moral distress as institutional constraint locates the phenomenon, and precisely there that evaluated interventions are scarcest. Such work should be anchored in the process model used here, should adopt validated moral-distress instruments in preference to surrogate measures, and should employ designs capable of controlling confounding, the criterion that emerged as the consistent methodological weakness of the existing corpus; measurement, in particular, will need to reach beyond individual experience if interventions pitched at the level of the organisation are to be captured at all. For leadership and education the corresponding caution is that interventions cannot yet be ranked on the strength of current evidence, and that structural and preventive options are more accurately regarded as under-evaluated than as ineffective. A scoping design cannot adjudicate effectiveness; but by rendering the contours of the evidence visible, it can direct the next generation of studies towards the questions that matter most.

## Figures and Tables

**Table 1:** Characteristics of the 32 included studies (primary coding); reference numbers are given in square brackets after the study.

#	Study [Ref.]	Country	Population	Design (MMAT Cat.)	Modality (Primary; Secondary)	Level	Point of Action (Primary; Secondary)	Outcome—Instrument
1	Abbasi et al. 2019 [21]	Iran	ICU nurses	RCT (2)	M1	L1	A2	MD direct—MDS
2	Allen & Butler 2016 [22]	USA	Critical care nurses	Mixed methods (5)	M1	L1	A2	MD direct—MDS-R
3	Beumer 2008 [23]	USA	ICU nurses	Non-randomised (3)	M1; (M6)	L1	A2; A3	Non-validated—author questionnaire
4	Bevan & Emerson 2020 [24]	USA	Critical care nurses	Mixed methods (5)	M1; M5	L2	A2	MD direct—MDS-R
5	Bosshardt et al. 2018 [25]	USA	Nurses (acute care)	Mixed methods (5)	M7	L3	A1	MD direct—MDS-R
6	Brandon et al. 2014 [26]	USA	Mixed: paediatric care providers	Non-randomised (3)	M3; M4/M2 (integrated)	L3	A2; A3	MD direct—modified MDS
7	Brännström et al. 2025 [27]	Sweden	Mixed: multi-professional ward staff	Cluster RCT (2)	M3	L2	A2	MD direct—MMD-HP, MDT
8	Browning & Cruz 2018 [28]	USA	ICU nurses	Non-randomised (3)	M2	L2	A3; A2	MD direct—MDS-R
9	Bruce & Allen 2020 [29]	USA	Oncology nurses	Non-randomised (3)	M1; M2 (integrated)	L2	A2; A3	MD direct—MDS-R
10	Chiafery et al. 2018 [30]	USA	ICU nurses	Mixed methods (5)	M3	L2	A2	MD direct—MDT
11	Davis & Batcheller 2020 [31]	USA	Mixed: critical care staff (majority nurses)	Non-randomised (3)	M6; M2/M3 (integrated)	L2	A3; A2	Surrogate—CD-RISC-25
12	Epstein et al. 2023 [32]	USA	Mixed: interprofessional clinicians	Mixed methods (5)	M3	L3	A2; A1	MD direct—MDT (+GES)
13	Fontenot & White 2019 [33]	USA	Critical care nurses	Non-randomised (3)	M2	L2	A3	MD direct—MDT
14	Hamric & Epstein 2017 [34]	USA	Mixed: interprofessional clinicians	Descriptive (4)	M3	L3	A2; A1	MD direct (partial)—MDT
15	Iaboni et al. 2022 [35]	Canada	Mixed: dementia/long-term care staff	Descriptive (4)	M7	L3	A2	Surrogate—acceptance/use survey
16	Jakobsen et al. 2023 [36]	Norway	Long-term care nurses	Mixed methods (5)	M5	L1	A3; A2	Surrogate—digital reflection register
17	Kälvemark Sporrong et al. 2007 [52]	Sweden	Mixed: physicians, nurses, assistants, pharmacists, dispensers	Non-randomised (3)	M1; M3 (integrated)	L2	A2	Surrogate—QWC subscale
18	Leggett et al. 2013 [37]	USA	Nurses (burn centre)	Non-randomised (3)	M1	L1	A2	MD direct—MDS-R
19	Lillemoen & Pedersen 2015 [38]	Norway	Mixed: community health-service staff	Qualitative (1)	M3	L2	A2	Surrogate/qualitative—group interviews
20	Meziane et al. 2018 [39]	Canada	Nurses (acute care, end-of-life)	Mixed methods (5)	M5; M2/M3	L2	A3; A2	MD direct—MDS-R
21	Molazem et al. 2013 [40]	Iran	Nurses	RCT (2)	M1	L1	A2	MD direct—MDS (Persian)
22	Monteverde 2016 [41]	Switzerland	Nursing students	Non-randomised (3)	M1	L1	A2	MD direct—MDT (vignettes)
23	Reilly & Jurchak 2017 [42]	USA	Nurses	Qualitative (1)	M3	L2	A2	Surrogate/qualitative—focus groups
24	Robinson et al. 2014 [43]	USA	Nurses (clinical ethics residency)	Mixed methods (5)	M1	L1	A2	MD direct—MDS-R
25	Rogers et al. 2008 [44]	USA	NICU nurses	Non-randomised (3)	M1	L1	A2	Surrogate—comfort scale (CLCDI)
26	Rushton et al. 2021 [45]	USA	Nurses	Non-randomised (3)	M1; M6	L1	A2; A3	MD direct—MDT (+scales)
27	Saeedi et al. 2019 [46]	Iran	ICU nurses	RCT (2)	M5	L1	A3	MD direct—MDS (Persian)
28	Tavakol et al. 2023 [47]	Iran	Nurses	Mixed methods (5)	M1; M7 (integrated)	L2	A2; A1	MD direct—MDS-R
29	Vaclavik et al. 2018 [48]	USA	Nurses	Non-randomised (3)	M6; M2 (integrated)	L2	A3	MD direct—MDS-R (single item)
30	van der Dam et al. 2011 [49]	Netherlands	Mixed: nursing-home care staff	Qualitative (1)	M3	L2	A2	Surrogate/qualitative—case analysis
31	Wocial et al. 2017 [50]	USA	PICU nurses	Non-randomised (3)	M4; M3	L2	A2	MD direct—MDS-R + MDT
32	Wocial et al. 2024 [51]	USA	Mixed: clinicians (majority nurses)	Mixed methods (5)	M3; M7 (integrated)	L2	A2; A3	MD direct—MDT

Note: #, row number; reference numbers are given in square brackets. MMAT category: 1, qualitative; 2, randomised controlled trial; 3, quantitative non-randomised; 4, quantitative descriptive; 5, mixed methods. Modality: M1, education; M2, facilitated case discussion and debriefing; M3, ethics deliberation and consultation services; M4, interprofessional rounds; M5, reflective and narrative methods; M6, resilience and mindfulness methods; M7, structural-organisational measures; integrated, components inseparable. Level: L1, individual; L2, team or unit; L3, organisation or system. Point of action: A1, preventive, at the source; A2, supportive-enabling, around the moral event; A3, processing-downstream. Outcome: MD direct, direct moral-distress measure; surrogate, adjacent construct. Abbreviations: CD-RISC-25, Connor–Davidson Resilience Scale; CLCDI, Comfort Level Communication with Dying Infants; GES, Global Empowerment Scale; ICU, intensive care unit; MDS, Moral-Distress Scale; MDS-R, Moral-Distress Scale—Revised; MDT, Moral-Distress Thermometer; MMD-HP, Measure of Moral Distress for Healthcare Professionals; NICU, neonatal intensive care unit; PICU, paediatric intensive care unit; QWC, Quality Work Competence; RCT, randomised controlled trial.

## Data Availability

All data supporting the findings of this review are contained within the article and its Supplementary Material. The review protocol, the coding framework and the full search strategy are openly available on the Open Science Framework (https://osf.io/akczg (accessed on 12 September 2026)).

## Supplementary Materials

The following supporting information can be downloaded at: https://www.mdpi.com/article/10.3390/healthcare14183023/s1. All supplementary material is contained in a single document comprising six sections: File S1, full electronic search strategies; File S2, PRISMA-ScR checklist; File S3, coding framework, category definitions and adjudication log; File S4, complete data charting table for the 32 included studies; File S5, sensitivity analysis of the evidence gap map under multiple coding; File S6, Reported direction of effect by study design and by the fourth MMAT criterion.
