# Supplementary material for: Interventions to Address Moral Distress in Nurses and Other Healthcare Professionals: A Scoping Review and Evidence Gap Map
Source: Healthcare (Basel). 2026 Sep 15;14(18):3023. doi: 10.3390/healthcare14183023 (PMC13606880; doi:10.3390/healthcare14183023)
Supplement: Supplementary file 1 [file healthcare-14-03023-s001.zip › healthcare-4536882-supplementary.pdf]

Supplementary Materials

This document contains all supplementary material accompanying the manuscript, in six sections. Throughout this document, included studies and methodological references are cited with the reference numbers of the main article in square brackets; included studies are additionally identified by first author and year.

- **Supplementary File S1.** Full electronic search strategies
- **Supplementary File S2.** PRISMA-ScR checklist
- **Supplementary File S3.** Coding framework, category definitions and adjudication log
- **Supplementary File S4.** Complete data charting table for the 32 included studies
- **Supplementary File S5.** Sensitivity analysis of the evidence gap map under multiple coding
- **Supplementary File S6.** Reported direction of effect by study design and by the fourth MMAT criterion

Supplementary File S1. Full electronic search strategies

Reported in accordance with PRISMA-S [16]. The search combined two concept blocks with AND: Block 1 (phenomenon) captured moral distress and its cognate constructs (moral residue, moral suffering, moral injury); Block 2 (population) captured health professionals of all groups and interprofessional teams. Free-text terms in title and abstract fields were used throughout; no controlled vocabulary was applied, because moral distress has no descriptor in MeSH, Emtree or the CINAHL Subject Headings. No intervention block was used; intervention status was determined at screening. No language, date or publication-status limits were applied. Databases were searched from inception to 13 July 2026 (all searches updated 14 July 2026). The strategies are reproduced below exactly as executed on each platform.

Databases and platforms

Ovid MEDLINE (1946 to 13 July 2026) — Ovid

Embase (1974 to 13 July 2026) — Ovid

Cochrane Central Register of Controlled Trials (CENTRAL) — Ovid (EBM Reviews)

Cochrane Database of Systematic Reviews (CDSR) — Ovid (EBM Reviews)

CINAHL — EBSCOhost

Web of Science Core Collection — Clarivate

PubMed — NLM

A.1 Ovid multi-file (MEDLINE, Embase, CENTRAL, CDSR)

1 (moral distress or moral distresses or morally distressed or morally distressing).ti,ab.
2 (moral residue or moral suffering).ti,ab.
3 (moral injury or morally injured).ti,ab.
4 or/1-3
5 nurs*.ti,ab.
6 (physician* or doctor* or clinician*).ti,ab.
7 (health care professional* or healthcare professional* or health professional*).ti,ab.
8 (health care worker* or healthcare worker* or health care provider* or healthcare provider*).ti,ab.
9 (health care staff or healthcare staff or medical staff or hospital staff or clinical staff or intensive care staff or ICU staff).ti,ab.
10 (midwif* or pharmacist* or chaplain* or social worker* or respiratory therapist*).ti,ab.
11 (allied health or interprofessional or interdisciplinary or care team*).ti,ab.
12 (resident physician* or medical resident* or medical student*).ti,ab.

13 or/5-12
14 4 and 13

A.2 CINAHL (EBSCOhost)

S1 TI ("moral distress" OR "moral distresses" OR "morally distressed"
OR "morally distressing" OR "moral residue" OR "moral suffering"
OR "moral injury" OR "morally injured")
OR AB (same terms)

S2 TI (nurs* OR physician* OR doctor* OR clinician* OR midwif*
OR pharmacist* OR chaplain* OR "health care professional*"
OR "healthcare professional*" OR "health professional*"
OR "health care worker*" OR "healthcare worker*"
OR "health care provider*" OR "healthcare provider*"
OR "medical staff" OR "hospital staff" OR "clinical staff"
OR "allied health" OR "social worker*" OR "respiratory therapist*"
OR "resident physician*" OR "medical resident*"
OR "medical student*" OR "care team*"
OR interprofessional OR interdisciplinary)
OR AB (same terms)

S3 S1 AND S2

Free-text only, consistent with the design; CINAHL Subject Headings are deliberately not used.

A.3 Web of Science Core Collection

TS=(("moral distress" OR "moral distresses" OR "morally distressed" OR "morally distressing" OR "moral residue" OR "moral suffering" OR "moral injury" OR "moral injuries" OR "morally injured") AND (nurs* OR physician* OR doctor* OR clinician* OR midwif* OR pharmacist* OR chaplain* OR "health care professional" OR "health care professionals" OR "healthcare professional" OR "healthcare professionals" OR "health professional" OR "health professionals" OR "health care worker" OR "health care workers" OR "healthcare worker" OR "healthcare workers" OR "health care provider" OR "health care providers" OR "healthcare provider" OR "healthcare providers" OR "health care staff" OR "healthcare staff" OR "medical staff" OR "hospital staff" OR "clinical staff" OR "allied health" OR interprofessional OR interdisciplinary OR "care team" OR "care teams" OR "resident physician" OR "resident physicians" OR "medical resident" OR "medical residents" OR "medical student" OR "medical students" OR "social worker" OR "social workers" OR "respiratory therapist" OR "respiratory therapists"))

Entered in the Advanced Search query box with the database selector set to Web of Science Core Collection. TS= additionally indexes author keywords and KeyWords Plus and is therefore not strictly equivalent to a title/abstract restriction.

A.4 PubMed

#1 "moral distress"[tiab] OR "moral distresses"[tiab] OR
"morally distressed"[tiab] OR "morally distressing"[tiab] OR
"moral residue"[tiab] OR "moral suffering"[tiab] OR
"moral injury"[tiab] OR "morally injured"[tiab]

#2 nurse[tiab] OR nurses[tiab] OR nursing[tiab] OR nurs*[tiab] OR
physician[tiab] OR physicians[tiab] OR doctor[tiab] OR doctors[tiab] OR
clinician[tiab] OR clinicians[tiab] OR
"healthcare professional"[tiab] OR "healthcare professionals"[tiab] OR
"health care professional"[tiab] OR "health care professionals"[tiab] OR
"health professional"[tiab] OR "health professionals"[tiab] OR
"healthcare worker"[tiab] OR "healthcare workers"[tiab] OR
"health care worker"[tiab] OR "health care workers"[tiab] OR
"healthcare provider"[tiab] OR "healthcare providers"[tiab] OR
"health care provider"[tiab] OR "health care providers"[tiab] OR
"healthcare staff"[tiab] OR "health care staff"[tiab] OR
"medical staff"[tiab] OR "hospital staff"[tiab] OR "clinical staff"[tiab] OR
"intensive care staff"[tiab] OR "ICU staff"[tiab] OR
"resident physician"[tiab] OR "resident physicians"[tiab] OR
"medical resident"[tiab] OR "medical residents"[tiab] OR
midwife[tiab] OR midwives[tiab] OR midwifery[tiab] OR
"allied health"[tiab] OR "social worker"[tiab] OR "social workers"[tiab] OR
"respiratory therapist"[tiab] OR "respiratory therapists"[tiab] OR
pharmacist[tiab] OR pharmacists[tiab] OR chaplain[tiab] OR chaplains[tiab] OR
"medical student"[tiab] OR "medical students"[tiab] OR
interprofessional[tiab] OR interdisciplinary[tiab] OR
"care team"[tiab] OR "care teams"[tiab]

#3 #1 AND #2

Records retrieved

PubMed 3,101; Ovid multi-file 6,421; CINAHL 1,908; Web of Science Core Collection 5,178. Total 16,608 records; 6,792 duplicates removed; 9,816 unique records screened on title and abstract; 128 retained for full-text assessment; 29 met the eligibility criteria; three further eligible reports were identified by forward and backward citation searching, giving 32 included studies.

Reference

16. Rethlefsen ML, Kirtley S, Waffenschmidt S, et al. PRISMA-S: an extension to the PRISMA Statement for reporting literature searches in systematic reviews. Syst Rev. 2021;10(1):39. doi:10.1186/s13643-020-01542-z

Supplementary File S2. PRISMA-ScR checklist

Section	Item	PRISMA-ScR checklist item	Reported in (section of the manuscript)
**TITLE**			
Title	1	Identify the report as a scoping review.	Title page. The title states "… a scoping review and evidence gap map"; the article type is designated "Scoping Review".
**ABSTRACT**			
Structured summary	2	Provide a structured summary that includes (as applicable): background, objectives, eligibility criteria, sources of evidence, charting methods, results, and conclusions that relate to the review questions and objectives.	Abstract (Background/Objectives; Methods; Results; Conclusions).
**INTRODUCTION**			
Rationale	3	Describe the rationale for the review in the context of what is already known. Explain why the review questions/objectives lend themselves to a scoping review approach.	Introduction, paragraphs 1–4. Justification of the scoping design: Section 2, paragraph 1.
Objectives	4	Provide an explicit statement of the questions and objectives being addressed with reference to their key elements (e.g., population or participants, concepts, and context) or other relevant key elements used to conceptualize the review questions and/or objectives.	Introduction, final paragraph (three mapping dimensions and the appraisal objective). Population, concept and context elements: Section 2.2.
**METHODS**			
Protocol and registration	5	Indicate whether a review protocol exists;	Section 2, paragraph 1: protocol registered on the Open Science

Section	Item	PRISMA-ScR checklist item	Reported in (section of the manuscript)
		state if and where it can be accessed (e.g., a Web address); and if available, provide registration information, including the registration number.	Framework (DOI 10.17605/OSF.IO/AKCZG). Data Availability Statement.
Eligibility criteria	6	Specify characteristics of the sources of evidence used as eligibility criteria (e.g., years considered, language, and publication status), and provide a rationale.	Section 2.2: population, concept and context, each operationalised; eligible designs and exclusions; no language, date or publication-status restriction. Rationale for the absence of an intervention search block: Section 2.1, paragraph 1.
Information sources	7	Describe all information sources in the search (e.g., databases with dates of coverage and contact with authors to identify additional sources), as well as the date the most recent search was executed.	Section 2.1, paragraph 2: PubMed, Ovid multi-file (MEDLINE, Embase, CENTRAL, CDSR), CINAHL (EBSCOhost) and the Web of Science Core Collection, searched from inception; most recent search 14 July 2026; forward and backward citation searching in July 2026.
Search	8	Present the full electronic search strategy for at least 1 database, including any limits used, such that it could be repeated.	Section 2.1, paragraph 1 (structure and design decisions). Supplementary File S1: full line-by-line strategies for all four platforms as executed, reported in accordance with PRISMA-S.
Selection of sources of evidence	9	State the process for selecting sources of evidence (i.e., screening and eligibility) included in the scoping review.	Section 2.6: two independent reviewers at both stages, inter-screener agreement 94%, disagreements resolved by consensus with a third reviewer available. Section 3.1: numbers at each stage.
Data charting process	10	Describe the methods of charting data from the included sources of	Section 2.4: charting form specified a priori, piloted on the first five studies and frozen

Section	Item	PRISMA-ScR checklist item	Reported in (section of the manuscript)
		evidence (e.g., calibrated forms or forms that have been tested by the team before their use, and whether data charting was done independently or in duplicate) and any processes for obtaining and confirming data from investigators.	before full application; fields listed. Section 2.5: coding of the three mapping dimensions. Section 2.6: independent duplicate screening, charting and coding reported as separate steps; Cohen's κ per mapping dimension (M 0.75, L 0.84, A 0.79); consensus resolution; software used. Supplementary Files S3 and S4.
Data items	11	List and define all variables for which data were sought and any assumptions and simplifications made.	Section 2.4: charted variables, including the TIDieR-aligned intervention description. Section 2.5: intervention modality (M1–M7), intervention level (L1–L3), point of action (A1–A3), dominance rule and cross-cutting tags. Supplementary File S3: operational definitions, delimitation criteria, anchor examples and adjudication log. Supplementary File S4: the charted dataset.
Critical appraisal of individual sources of evidence	12	If done, provide a rationale for conducting a critical appraisal of included sources of evidence; describe the methods used and how this information was used in any data synthesis (if appropriate).	Section 2.3: rationale for appraising within a scoping review; MMAT version 2018 at item level; no aggregate score; no exclusion, ranking or weighting on quality grounds.
Synthesis of results	13	Describe the methods of handling and summarizing the data that were charted.	Section 2.7: marginal frequencies, two cross-tabulations, and the joint display of all three dimensions as an evidence gap map, with bubble area and ring encoding; accompanying narrative synthesis. Sensitivity analysis under multiple coding

Section	Item	PRISMA-ScR checklist item	Reported in (section of the manuscript)
			(Supplementary File S5) and tabulation of reported direction of effect by study design (Supplementary File S6).
**RESULTS**			
Selection of sources of evidence	14	Give numbers of sources of evidence screened, assessed for eligibility, and included in the review, with reasons for exclusions at each stage, ideally using a flow diagram.	Section 3.1: 16,608 records identified, 6,792 duplicates removed, 9,816 screened, 128 full texts assessed, 29 included, 3 added by citation searching, 32 included in total. Figure 1: PRISMA-ScR flow diagram with the reasons for exclusion at the full- text stage.
Characteristics of sources of evidence	15	For each source of evidence, present characteristics for which data were charted and provide the citations.	Table 1: characteristics of the 32 included studies with citations [21–52]. Section 3.2: country of origin, professional composition and MMAT design category. Supplementary File S4, Panel A: the full charted characteristics.
Critical appraisal within sources of evidence	16	If done, present data on critical appraisal of included sources of evidence (see item 12).	Section 3.7 and Figure 3: item-level MMAT ratings for every included study as a traffic-light plot.
Results of individual sources of evidence	17	For each included source of evidence, present the relevant data that were charted that relate to the review questions and objectives.	Table 1: primary coding (modality, level, point of action, outcome construct) per study. Section 3.8: reported direction of effect. Supplementary File S4, Panel B: the study-level codes and reported directions.
Synthesis of results	18	Summarize and/or present the charting results as they relate to the review questions and objectives.	Sections 3.3 to 3.6 and 3.8; Figure 2: integrated evidence gap map of modality against point of action, stratified by intervention level.
**DISCUSSION**			
Summary of evidence	19	Summarize the main results (including an	Section 4.1 (principal findings), Section 4.2 (relation to previous

Section	Item	PRISMA-ScR checklist item	Reported in (section of the manuscript)
		overview of concepts, themes, and types of evidence available), link to the review questions and objectives, and consider the relevance to key groups.	evidence) and Section 4.3 (why the evidence is skewed).
Limitations	20	Discuss the limitations of the scoping review process.	Section 4.4 (strengths and limitations), second and third paragraphs, including the contingency of the observed gaps on the eligibility criteria and the coding framework.
Conclusions	21	Provide a general interpretation of the results with respect to the review questions and objectives, as well as potential implications and/or next steps.	Section 5 (conclusions), including the conceptual and practical contribution and the research agenda derived from the empty cells of the map.
**FUNDING**			
Funding	22	Describe sources of funding for the included sources of evidence, as well as sources of funding for the scoping review. Describe the role of the funders of the scoping review.	Funding statement (back matter): the scoping review received no external funding and no funder had any role in its design, conduct or reporting; the funding of the included sources of evidence was not charted and is therefore not reported.

Abbreviations: MMAT, Mixed Methods Appraisal Tool; PRISMA-ScR, Preferred Reporting Items for Systematic reviews and Meta-Analyses extension for Scoping Reviews; PRISMA-S, PRISMA extension for reporting literature searches; TIDieR, Template for Intervention Description and Replication.

Checklist reproduced from [15]: Tricco AC, Lillie E, Zarin W, O'Brien KK, Colquhoun H, Levac D, et al. PRISMA Extension for Scoping Reviews (PRISMA-ScR): checklist and explanation. *Ann Intern Med.* 2018;169(7):467–473. doi:10.7326/M18-0850.

Supplementary File S3. Coding framework, category definitions and adjudication log

1. Purpose and scope

This document reports, in full, the framework by which the included intervention studies were coded on the three mapping dimensions of the review. It is provided so that every classification underlying the evidence gap map can be reconstructed, inspected and, where necessary, contested.

The scheme follows a best-fit framework synthesis [20]: a category system derived a priori from the convergent terminology of the relevant prior syntheses [5–8] was applied deductively and extended inductively where studies did not fit. It serves the mapping of the field and not the assessment of effectiveness.

Coding was applied to the 32 included intervention studies only. The basis of every assignment was the full text, together with companion publications reporting the same programme. Information that could not be established with confidence from the full text was recorded as such and was not supplied from other sources.

2. Principles of coding

The unit of coding is the intervention, or its component, and not the study as a whole. Each study received a mandatory assignment on three dimensions together with the cross-cutting tags:

- **Dimension 1 (modality):** primary code, mutually exclusive, assigned under the dominance rule; an optional secondary modality was recorded in a separate field.
- **Dimension 2 (intervention level):** one value per study.
- **Dimension 3 (point of action in the moral-distress process):** dominant point of action mandatory, secondary optional.
- **Cross-cutting tags:** multi-valued; they cut across the dimensions and were coded independently. Empowerment was deliberately treated as a tag and not as a modality, because it is realised through several intervention forms.

3. Decision rule and dominance rule

The primary modality is the component that predominates in time and in substance. Where two or more components are inseparably interlocked without one clearly predominating, the flag “integrated programme” was set; the primary modality was then assigned by the component with the largest session or time share and, at genuine parity, by the principal aim stated by the authors. Secondary and hybrid components were always documented in the field “secondary modality”, so that elements contained within a bundle remain visible and do not disappear into a collective cluster.

4. Dimension 1: intervention modality (M)

The categories adopt the terminology on which the prior syntheses converge [5–8], so that the map connects directly to that work.

Category	Definition	Delimitation	Anchor examples
**M1 Education (ethics / intervention)**	Structured transmission of knowledge and skills concerning moral distress, ethical analysis and strategies for action; didactic, where applicable with activating elements (case work, role play, simulation, critical-dialogical pedagogy).	Where ethicist-supported analysis of a current case is central: M3. Where the session serves primarily emotional processing: M2.	Molazem [40] (4A training); Robinson [43] (clinical ethics residency); Bevan [24] (problem-posing, empowerment)
**M2 Facilitated case discussion and debriefing**	Recurrent, moderated group sessions for the retrospective and predominantly emotional processing of distressing situations; moderation usually by social work, chaplaincy or nursing leadership.	Ethicist-supported discussion aimed at ethical analysis: M3. Interprofessional round tied to treatment goals: M4.	Fontenot & White [33]; Browning & Cruz [28]
**M3 Ethics deliberation and consultation services**	Ethicist- or ethics-team-supported structured deliberation of ethical cases and of the sources of moral distress; either as an on-demand service or as a recurrent format.	Emotional processing without ethical analysis: M2. Pure transmission of knowledge: M1.	Epstein [32] (moral distress consultation service); van der Dam [49] (moral case deliberation); Chiafery [30] (ethics huddles)
**M4 Interprofessional rounds**	Regular interprofessional case discussions tied to individual patients and their treatment goals, with an ethical and a communicative	Uniprofessional, ethicist-led case discussion: M3.	Wocial [50] (PEACE rounds)

Category	Definition	Delimitation	Anchor examples
	component.		
**M5 Reflective and narrative methods**	Individual or minimally facilitated reflective or expressive procedures in which the professional writes down or reflects upon their own experience in a structured way.	Moderated group processing: M2. Collective-dialogical pedagogy: M1.	Saeedi [46] (narrative writing); Meziane [39] (structured reflection); Jakobsen [36] (digital reflection tool)
**M6 Resilience and mindfulness methods**	Procedures aimed at strengthening individual coping and recovery (mindfulness or MBSR, resilience training, yoga, self-care elements).	Where the study measures resilience only, without reference to moral distress, this is recorded in the outcome tag as a surrogate. Where such elements form one component of a predominantly educational curriculum, they are coded as a secondary modality (see Rushton in the adjudication log).	Vaclavik [48] (MBSR bundle); Davis [31] (resilience bundle)
**M7 Structural-organisational measures**	Interventions in rules, responsibilities, decision-making structures or tools that alter the action-constraining conditions themselves.	Individual competence development: M1. Cognitive decision aids are differentiated by their point of action (see Iaboni [35] in the adjudication log).	Bosshardt [25] (shift of competence by policy); Iaboni [35] (practice and decision aid)

Flag "integrated programme": to be set where several components act inseparably together. Examples: Davis [31] (M6 primary; M2/M3 secondary), Brandon [26] (M3 primary; M4/M2 secondary), Bruce & Allen [29] (M1 primary; M2 secondary), Kälvemark Sporrong [52] (M1 primary; M3 secondary, hybrid), Tavakol [47] (M1 primary; M7 secondary), Wocial 2024 [51] (M3 primary; M7 secondary).

5. Dimension 2: intervention level (L)

Conceptually anchored in Jameton's account of moral distress [1] as a consequence of external, institutional constraints on action, and in the observation, emphasised by Amos and Epstein [8], that measures at the organisational level are rare.

Value	Definition and assignment criterion	Examples
**L1 Individual**	The intervention is directed at the individual professional and operates through their competence, reflection or coping.	Saeedi [46]; Molazem [40]; Vaclavik [48]; Monteverde [41]
**L2 Team / unit**	Group- or ward-related process involving several members of one unit.	Wocial 2017 [50]; Chiafery [30]; Browning & Cruz [28]; van der Dam [49]; Brännström [27]
**L3 Organisation / system**	Institution-wide service, rule or structural change.	Epstein [32] (consultation service); Bosshardt [25] (policy); Iaboni [35] (toolkit introduced organisation-wide)

Rule: the level coded is the level at which the intervention is delivered and structurally anchored, not the level at which the cause of the moral distress lies. Where a measure spans several levels, the highest structurally anchored level is coded and the span is recorded in free text.

6. Dimension 3: point of action in the moral-distress process (A)

This analytically load-bearing dimension operationalises the moral distress model of Morley, Bradbury-Jones and Ives [12], which was developed expressly in order to locate interventions along the course of distress. It renders codable the individual-centred and aftercare-heavy skew of the field.

Value	Definition	Examples
**A1 At the source (preventive)**	Reduces or removes the triggering, action-constraining condition before moral distress arises.	Bosshardt [25]
**A2 At the initial distress (supportive-enabling)**	Strengthens, in the temporal vicinity of the moral event, perception, ethical competence, capacity to act and shared sense-making.	Education; deliberation; huddles; rounds; consultation service (barriers and strategies)
**A3 At the reactive distress (processing-downstream)**	Addresses the distress persisting after the event and the moral residue.	Debriefing; narrative writing; resilience and mindfulness methods

Rule: multiple coding is permitted; the dominant point of action is recorded first, the secondary one thereafter. Examples: Rushton [45] A2 + A3; Epstein consultation service [32] A2 (and A1, in so far as barriers to addressing causes are named).

7. Cross-cutting tags (facets)

The facets are aligned with the TIDieR checklist [19], so that the description of the interventions rests on a documented and citable basis and the assignment of modality remains traceable.

Tag	Permitted values / format	Coding rule (TIDieR reference)
**T1 Empowerment / moral agency**	yes | no, with brief justification	"yes" where the strengthening of moral agency is a declared aim, irrespective of modality. Examples coded yes: Abbasi [21]; Bevan [24]; Bosshardt [25]; Epstein [32]; Hamric & Epstein [34]; Tavakol [47]; Wocial 2024 [51].
**T2 Facilitation profile**	self-directed | peer | interprofessional | ethicist or ethics team | chaplaincy | social work | nursing or ward leadership | authors	who provided the intervention
**T3 Dose / duration**	single session | longitudinal; with number, frequency, total duration (free text)	extent and temporal structure (when and how much)
**T4 Trigger**	universal or planned | event- or occasion-related	trigger for delivery (what procedures)
**T5 Embedding**	add-on activity | integrated into the workflow	anchoring in everyday work (where / tailoring); a proxy for sustainability
**T6 Outcome construct**	direct moral distress (MDS / MDS-R / MMD-HP / MDT) | surrogate (construct to be named: resilience, empowerment, competence, comfort)	"direct" only where a validated moral-distress instrument is used; otherwise surrogate
**T7 Theoretical framework**	none named | named (free text)	for example 4A, Nathaniel, Freire, Johns, democratic dialogue

8. Standing adjudication log of borderline decisions

These decisions were recorded before full coding and were carried forward during it. Together with the definitions above they constitute the operational basis of the map.

Study	Contested between	Decision	Rationale
**Rushton [45] (MEPRA)**	M1 vs M6	primary M1, secondary M6; L1; A2 and A3	Revised on full-text review: the intervention is an educational curriculum delivered as an academy, of which mindfulness and resilience training form one component among several; the didactic frame therefore predominates.

Study	Contested between	Decision	Rationale
**Bevan [24]**	M1 vs M5	primary M1, secondary M5; L2; empowerment yes	Reported in the literature as problem-posing education; collective pedagogy, not an individually expressive procedure.
**Kälvemark Sporrong [52]**	M1 vs M3	primary M1, secondary M3; hybrid	The lecture component provides the frame, ethics rounds supplement it.
**Bruce & Allen [29]**	M1 vs M2	primary M1, secondary M2; integrated	Two of three components are educational (communication, end-of-life care).
**Davis [31]**	M6 vs integrated programme	primary M6; integrated; secondary M2/M3	The resilience bundle predominates; outcome is a surrogate (CD-RISC).
**Brandon [26]**	M3 vs M4	primary M3, secondary M4/M2; integrated; L3	A specialised quality-of-life consultation forms the core, supplemented by rounds and debriefs.
**Iaboni [35]**	M7 structural vs cognitive aid	modality M7, but point of action A2	The toolkit operates as decision support, not as a change of structure; outcome is a surrogate (acceptance).
**Meziane [39]**	M5 vs M3	primary M5	Individual written reflection predominates; group discussion is secondary.
**Epstein [32] / Hamric [34] (consultation service)**	level L2 vs L3	L3; M3; A2 (and A1)	An institution-wide service, not a ward-internal one.
**Tavakol [47]**	M1 vs M7	primary M1, secondary M7; integrated; L2; A2 (and A1)	The educational programme predominates; ward-level management meetings addressing workload and task allocation act at the source and are coded as the secondary point of action.
**Wocial 2024 [51]**	M3 vs M7	primary M3, secondary M7; integrated; L2; A2 (and A3)	Ethics conversations form the core; the accompanying structural element is subordinate.
**Brännström [27]**	L2 vs L3	L2; M3; A2	Ethics communication groups are delivered and anchored at ward

Study	Contested between	Decision	Rationale
			level, although the trial randomised at the level of the unit.

The complete charted dataset to which this framework was applied is reported in Supplementary File S4.

9. Coding process and quality assurance

The scheme was first piloted on five to six studies and refined there; it was then frozen and only afterwards applied in full. All entries were coded independently in duplicate; inter-rater agreement was computed per dimension as Cohen's kappa on the final dataset of 32 studies and was 0.75 for modality (M), 0.84 for level (L) and 0.79 for point of action (A). Discrepancies were resolved by consensus and, where they persisted, by a third person; the decisions were carried forward in the adjudication log. The reporting of the charting process follows PRISMA-ScR [15] (item "data charting process").

10. Extraction grid (fields of the working workbook)

Field	Values / format
Modality_primary	M1 ∣ M2 ∣ M3 ∣ M4 ∣ M5 ∣ M6 ∣ M7
Modality_secondary	M1 … M7 ∣ (empty)
Flag_integrated	yes ∣ no
Level	L1 ∣ L2 ∣ L3
Point_of_action_primary	A1 ∣ A2 ∣ A3
Point_of_action_secondary	A1 ∣ A2 ∣ A3 ∣ (empty)
Tag_empowerment	yes ∣ no (with justification)
Facilitation profile	controlled vocabulary (T2)
Dose	single session ∣ longitudinal (with free text)
Trigger	universal ∣ occasion-related
Embedding	add-on ∣ integrated
Outcome_construct	direct moral distress ∣ surrogate (with instrument)
Theoretical framework	none ∣ free text
Coder_1 / Coder_2 / consensus / comment	free text

References cited in this supplementary document (numbered as in the reference list of the main article)

1. Jameton, A. *Nursing Practice: The Ethical Issues*; Prentice-Hall: Englewood Cliffs, NJ, 1984.

5. Morley, G.; Field, R.; Horsburgh, C.C.; Burchill, C. Interventions to Mitigate Moral Distress: A Systematic Review of the Literature. *Int. J. Nurs. Stud.* **2021**, *121*, 103984, doi:10.1016/j.ijnurstu.2021.103984.

6. Emami Zeydi, A.; Ghazanfari, M.J.; Suhonen, R.; Adib-Hajbaghery, M.; Karkhah, S. Effective Interventions for Reducing Moral Distress in Critical Care Nurses. *Nurs. Ethics* **2022**, *29*, 1047–1065, doi:10.1177/09697330211062982.

7. Imbulana, D.I.; Davis, P.G.; Prentice, T.M. Interventions to Reduce Moral Distress in Clinicians Working in Intensive Care: A Systematic Review. *Intensive Crit. Care Nurs.* **2021**, *66*, 103092, doi:10.1016/j.iccn.2021.103092.

8. Amos, V.K.; Epstein, E. Moral Distress Interventions: An Integrative Literature Review. *Nurs. Ethics* **2022**, *29*, 582–607, doi:10.1177/09697330211035489.

12. Morley, G.; Bradbury-Jones, C.; Ives, J. The Moral Distress Model: An Empirically Informed Guide for Moral Distress Interventions. *J. Clin. Nurs.* **2022**, *31*, 1309–1326, doi:10.1111/jocn.15988.

15. Tricco, A.C.; Lillie, E.; Zarin, W.; O'Brien, K.K.; Colquhoun, H.; Levac, D.; Moher, D.; Peters, M.D.J.; Horsley, T.; Weeks, L.; et al. PRISMA Extension for Scoping Reviews (PRISMA-ScR): Checklist and Explanation. *Ann. Intern. Med.* **2018**, *169*, 467–473, doi:10.7326/M18-0850.

19. Hoffmann, T.C.; Glasziou, P.P.; Boutron, I.; Milne, R.; Perera, R.; Moher, D.; Altman, D.G.; Barbour, V.; Macdonald, H.; Johnston, M.; et al. Better Reporting of Interventions: Template for Intervention Description and Replication (TIDieR) Checklist and Guide. *BMJ* **2014**, *348*, g1687, doi:10.1136/bmj.g1687.

20. Carroll, C.; Booth, A.; Leaviss, J.; Rick, J. "Best Fit" Framework Synthesis: Refining the Method. *BMC Med. Res. Methodol.* **2013**, *13*, 37, doi:10.1186/1471-2288-13-37.

Supplementary File S4. Complete data charting table

All 32 included studies, charted on the form frozen after piloting. Panel A reports the study and intervention characteristics extracted in accordance with the TIDieR checklist [19]; Panel B reports the codes assigned on the three mapping dimensions, the cross-cutting empowerment tag, the outcome construct and the reported direction of effect. Modality: M1 education; M2 facilitated case discussion and debriefing; M3 ethics deliberation and consultation services; M4 interprofessional rounds; M5 reflective and narrative methods; M6 resilience and mindfulness methods; M7 structural-organisational measures. Level: L1 individual; L2 team or unit; L3 organisation or system. Point of action: A1 preventive, at source; A2 supportive-enabling, around the moral event; A3 processing-downstream, reactive residue. Operational definitions, delimitation criteria and the adjudication log are reported in Section S3. The direction of effect is charted descriptively and does not constitute an assessment of effectiveness.

Panel A. Study and intervention characteristics

	Study [ref.]	Country	Population	Study design	Provider / facilitation profile	Dose and duration	Trigger	Embedding	Theoretical framework
1	Abbasi et al. 2019 [21]	Iran	ICU nurses	RCT (2-arm parallel)	researchers	block (2 days)	universal	add-on	Nathaniel (Moral Reckoning)
2	Allen & Butler 2016 [22]	USA	Critical care nurses	Mixed methods (survey + focus groups + one-group pre/post)	researchers	single session (2 h)	universal	add-on	none
3	Beumer 2008 [23]	USA	ICU nurses	Non-randomised, controlled (quasi- experimental), pre/post	interprofessional (leadership/EAP/CNS)	longitudinal (5 sessions/4 wks)	universal	add-on	4A
4	Bevan & Emerson 2020 [24]	USA	Critical care nurses	Mixed methods (one- group pre/post + thematic analysis)	researchers (Freirean-trained)	longitudinal (3 sessions)	universal	add-on	Freire; Young
5	Bosshardt et al. 2018 [25]	USA	Nurses (acute care)	Mixed methods (repeated cross- sectional survey + thematic analysis)	organisational (no facilitation)	permanent (structural)	universal	integrate d	none
6	Brandon et al. 2014 [26]	USA	Mixed: paediatric care providers	Quasi-experimental, repeated cross- sectional (unpaired pre/post)	interprofessional	longitudinal (programme)	universal/ scheduled	integrate d	family-centred QoL model
7	Brännström et al. 2025 [27]	Sweden	Mixed: multi- professional ward staff	Cluster RCT (open, non-blinded)	peer (trained ward ethics representatives)	longitudinal (monthly/6 mo)	scheduled	integrate d	Habermas (communicative action)
8	Browning & Cruz 2018 [28]	USA	ICU nurses	Non-randomised, controlled (non- equivalent control), pre/post	social work	longitudinal (monthly/6 mo)	scheduled	add-on	4A

	Study [ref.]	Country	Population	Study design	Provider / facilitation profile	Dose and duration	Trigger	Embedding	Theoretical framework
9	Bruce & Allen 2020 [29]	USA	Critical care nurses	One-group pre/post (quasi-experimental)	chaplaincy; nursing (CNS)	longitudinal (3-part)	universal	add-on	none
10	Chiafery et al. 2018 [30]	USA	ICU nurses	Mixed methods (one- group pre/post MDT + content analysis)	ethicist/ethics team	longitudinal (multiple)	event-triggered	integrate d	none
11	Davis & Batcheller 2020 [31]	USA	Mixed: critical-care staff (majority nurses)	One-group pre/post (quasi-experimental)	interprofessional/mi xed	longitudinal (6 mo)	universal	integrate d	none
12	Epstein et al. 2023 [32]	USA	Mixed: interprofessional clinicians	Mixed methods, quasi-experimental (pre/post with organic control + interviews)	ethicist/ethics team	on-demand (single consult)	event-triggered	integrate d	none
13	Fontenot & White 2019 [33]	USA	Critical care nurses	One-group pre/post pilot (quasi- experimental)	social work	longitudinal (biweekly/10 wks)	scheduled	add-on	none
14	Hamric & Epstein 2017 [32]	USA	Mixed: interprofessional clinicians	Descriptive programme developmen t/evaluation	ethicist/ethics team	on-demand (single consult)	event-triggered	integrate d	none
15	Jakobsen et al. 2023 [36]	Norway	Long-term care nurses	Mixed methods (sequential explanatory)	self-directed (digital)	longitudinal (per shift/6 wks)	universal	integrate d	none
16	Iaboni et al. 2022 [35]	Canada	Mixed: dementia / long-term care staff	Quantitative descriptive (cross- sectional survey)	self-directed/organi sational	permanent (toolkit)	event-triggered	integrate d	person-centred decision-making model
17	Leggett et al. 2013 [37]	USA	Nurses (burn centre)	Quasi-experimental (separate-sample pre/post); formative qualitative phase	researchers	longitudinal (4 weekly)	universal	add-on	none
18	Lillemoen & Pedersen 2015 [38]	Norway	Mixed: community health-service staff	Qualitative (focus groups, content analysis)	ethicist/ethics team	longitudinal (we ekly/biweekly)	scheduled	integrate d	Centre for Medical Ethics method
19	Meziane et al. 2018 [39]	Canada	Nurses (acute care, end-of-life)	Mixed methods, quasi-experimental (pre/post + thematic analysis)	nursing (palliative CNS)	longitudinal (3 sessions)	scheduled	add-on	Johns (structured reflection)
20	Molazem et al. 2013 [40]	Iran	Nurses	RCT (2-arm parallel, permuted blocks)	researchers	longitudinal (2 sessions/2 wks)	universal	add-on	4A
21	Monteverde 2016 [41]	Switzerl and	Nursing students	Quasi-experimental, within-subject repeated measures	researchers	single session (30 min)	universal	add-on	moral resilience
22	Reilly & Jurchak 2017 [42]	USA	Nurses	Qualitative (focus groups, thematic analysis)	ethicist (nurse- ethicist)	longitudinal (2×/mo/9 mo)	scheduled	add-on	none
23	Robinson et al. 2014 [43]	USA	Nurses (clinical ethics residency)	Mixed methods, quasi-experimental (pre/post + qualitative)	researchers/interpro fessional	longitudinal (98 h/10 mo)	universal	add-on	Rest (four- component)

	Study [ref.]	Country	Population	Study design	Provider / facilitation profile	Dose and duration	Trigger	Embedding	Theoretical framework
24	Rogers et al. 2008 [44]	USA	NICU nurses	One-group pre/post (quasi-experimental)	researchers	longitudinal (6 monthly)	universal	add-on	none
25	Rushton et al. 2021 [45]	USA	Nurses	Non-randomised, controlled (comparison group, ANCOVA)	interprofessional/re searchers	longitudinal (6 sessions/24 h)	universal	add-on	moral resilience (MEPRA)
26	Saeedi et al. 2019 [46]	Iran	ICU nurses	RCT (single-blind, 2-arm parallel)	self-directed	longitudinal (weekly/8 wks)	universal	add-on	none
27	Kälvemark Sporrong et al. 2007 [52]	Sweden	Mixed: physicians, nurses, assistants, pharmacists, dispensers	Non-randomised, controlled prospective	interprofessional/re searchers	longitudinal	scheduled	add-on	democratic dialogue
28	Tavakol et al. 2023 [47]	Iran	Nurses	Mixed methods, multiphase (one- group pre/post + qualitative phases)	researchers (ethicists + nursing academics)	longitudinal (3 mo, multi- component)	universal	add-on	Ewles & Simnett (programme planning)
29	Vaclavik et al. 2018 [48]	USA	Nurses	One-group pre/post (quasi-experimental)	interprofessional/mi xed	longitudinal	universal	integrate d	MBSR
30	van der Dam et al. 2011 [49]	Netherla nds	Mixed: nursing-home care staff	Qualitative (natural istic/responsive evaluation)	ethicist/ethics team	longitudinal	event-triggered	add-on	Moral Case Deliberation
31	Wocial et al. 2017 [50]	USA	PICU nurses	Quasi-experimental, pre/post with historical controls	ethicist + interprofessional	longitudinal (weekly/12 mo)	scheduled	integrate d	none
32	Wocial et al. 2024 [51]	USA	Mixed: clinicians (majority nurses)	Mixed methods (cross-over quasi- experimental + qualitative descriptive)	ethicist/ethics team	longitudinal (up to 9 FECs/3 wks)	scheduled	add-on	Wocial & Weaver definition; FEC format per Helft et al. 2009

Panel B. Coding on the three mapping dimensions, outcome and reported direction of effect

	Study [ref.]	M prim.	M sec.	Integ r.	L	A prim.	A sec.	Empowerment tag	Outcome construct	Instrument	Reported direction of effect	Comment
1	Abbasi et al. 2019 [21]	M1	–	no	L1	A2	–	yes (moral empowerment (Nathaniel))	MD direct	MDS	Reduction — OCR: conclusion, MD reduced	control arm received brief intervention
2	Allen & Butler 2016 [22]	M1	–	no	L1	A2	–	no	MD direct	MDS-R	Measured, not evaluable — MMAT: no inferential statistics possible	blended learning
3	Beumer 2008 [23]	M1	(M6)	no	L1	A2	A3	no	MD direct (non-validated)	author-developed questionnaire	Measured, not evaluable — direct but non- validated	self-care component; unit action plans

	Study [ref.]	M prim.	M sec.	Integr.	L	A prim.	A sec.	Empowerment tag	Outcome construct	Instrument	Reported direction of effect	Comment
											author- developed instrument	
4	Bevan & Emerson 2020 [24]	M1	M5	no	L2	A2	–	yes (Freirean consci entisation/empowerme nt)	MD direct	MDS-R (+PES, CWEQ- II)	Mixed — MMAT: total MD down, intensity up	borderline: form over aim → M1
5	Bosshardt et al. 2018 [25]	M7	–	no	L3	A1	–	yes (authority shift: nurses may order consults independently)	MD direct	MDS-R	Mixed — OCR: significant on 4 MDS-R items	only clearly preventive- structural case
6	Brandon et al. 2014 [26]	M3	M4/M2	yes	L3	A2	A3	no	MD direct	modified MDS	Mixed — OCR: most subscales similar, WQoL down	integrated programme
7	Brännström et al. 2025 [27]	M3	–	no	L2	A2	–	no	MD direct	MMD-HP + MDT (Swedish; + SwECQ)	No change — cluster RCT; no between-group change	interprofessional ward teams incl. nurses (nurse share not reported); identified via citation searching (July 2026)
8	Browning & Cruz 2018 [28]	M2	–	no	L2	A3	A2	no	MD direct	MDS-R	No change — OCR: pre- post not significant	case-based reflective debriefing
9	Bruce & Allen 2020 [29]	M1	M2	yes	L2	A2	A3	no	MD direct	MDS-R	No change — MMAT: MD unchanged at 8 months	MD increased post; integrated
10	Chiafery et al. 2018 [30]	M3	–	no	L2	A2	–	no	MD direct	MDT	Reduction — MMAT: significant MDT decrease	Nursing Ethics Huddles
11	Davis & Batcheller 2020 [31]	M6	M2/M3	yes	L2	A3	A2	no	Surrogate	CD-RISC-25 (resilience)	No direct MD estimate — resilience surrogate (CD-RISC)	integrated resilience bundle; no MD instrument
12	Epstein et al. 2023 [32]	M3	–	no	L3	A2	A1	yes (MDC identifies barriers and strategies; GES measured)	MD direct	MDT (+GES)	Reduction — MMAT: significant MDT decrease	institution-wide service
13	Fontenot & White 2019 [33]	M2	–	no	L2	A3	–	no	MD direct	MDT	No change — OCR: MDT did not change significantly	–
14	Hamric & Epstein 2017 [32]	M3	–	no	L3	A2	A1	yes (MDC: barriers/strategies)	MD direct (partial)	MDT (2015-16) + interviews	Measured, not evaluable — MMAT: evaluation only emergent	–
15	Jakobsen et al.	M5	–	no	L1	A3	A2	no	Surrogate	digital reflection register (non-	No direct MD estimate — surrogate	no validated MD instrument

	Study [ref.]	M prim.	M sec.	Integr.	L	A prim.	A sec.	Empowerment tag	Outcome construct	Instrument	Reported direction of effect	Comment
	2023 [36]									validated)	digital register	
16	Iaboni et al. 2022 [35]	M7	–	no	L3	A2	–	no	Surrogate	acceptance/use survey	No direct MD estimate — cross-sectional survey, no pre/post	borderline: structural in form, but cognitive aid → A2
17	Leggett et al. 2013 [37]	M1	–	no	L1	A2	–	no	MD direct	MDS-R (+self-efficacy)	Increase — OCR: higher MD in Group B post	–
18	Lillemoen & Pedersen 2015 [38]	M3	–	no	L2	A2	–	no	Surrogate/qualitative	group interviews	No direct MD estimate — qualitative	–
19	Meziane et al. 2018 [39]	M5	M2/M3	no	L2	A3	A2	no	MD direct	MDS-R	Mixed — MMAT: quant null, qual positive	individual reflection within group sessions
20	Molazem et al. 2013 [40]	M1	–	no	L1	A2	–	no	MD direct	MDS (Persian)	Reduction — OCR: significant reduction	–
21	Monteverde 2016 [41]	M1	–	no	L1	A2	–	no	MD direct	MDT (vignettes)	Reduction — 3 of 4 vignettes: significant reduction	students; shortest intervention
22	Reilly & Jurchak 2017 [42]	M3	–	no	L2	A2	–	no	Surrogate/qualitative	focus groups	No direct MD estimate — qualitative	–
23	Robinson et al. 2014 [43]	M1	–	no	L1	A2	–	no	MD direct	MDS-R (+ethics knowledge/self-efficacy)	Reduction — MMAT: significant MD decrease	intensive residency format
24	Rogers et al. 2008 [44]	M1	–	no	L1	A2	–	no	Surrogate	comfort scale (CLCDI)	No direct MD estimate — surrogate comfort scale	no MD instrument
25	Rushton et al. 2021 [45]	M1	M6	no	L1	A2	A3	no	MD direct	MDT (+ multiple scales)	No change — MMAT: no significant change	revised: educational curriculum (academy) → M1 primary; mindfu lness/resilience one of several components → M6 secondary
26	Saeedi et al. 2019 [46]	M5	–	no	L1	A3	–	no	MD direct	MDS (Persian)	No change — OCR: no effect on reducing MD	–
27	Kälvemark Sporrong et al. 2007 [52]	M1	M3	yes (hybrid)	L2	A2	–	no	Surrogate	QWC subscale (not MDS-comparable)	No direct MD estimate — surrogate QWC subscale	hybrid: lecture + ethics rounds
28	Tavakol et al.	M1	M7	yes	L2	A2	A1	yes (moral empowerment	MD direct	MDS-R (Persian)	Reduction —	secondary A1 via intra-ward management

	Study [ref.]	M prim.	M sec.	Integr.	L	A prim.	A sec.	Empowerment tag	Outcome construct	Instrument	Reported direction of effect	Comment
	2023 [47]							(explicit aim; qualitative theme))			uncontrolled one-group design; MDS-R	meetings addressing workload/task allocation at source; same group as Molazem 2013 but distinct programme; identified via citation searching (July 2026)
29	Vaclavik et al. 2018 [48]	M6	M2	yes	L2	A3	–	no	MD direct (single item)	MDS-R (single item only)	Reduction — OCR: significant decrease MDS-R freq.	mindfulness bundle
30	van der Dam et al. 2011 [49]	M3	–	no	L2	A2	–	no	Surrogate/qualitative	inductive case analysis	No direct MD estimate — qualitative	–
31	Wocial et al. 2017 [50]	M4	M3	no	L2	A2	–	no	MD direct	MDS-R + MDT	No change — MMAT: no significant change	PEACE Rounds
32	Wocial et al. 2024 [51]	M3	M7	yes	L2	A2	A3	yes (moral agency (explicit aim; mMAS))	MD direct	MDT (+ mMAS, mHECS)	No change — no between- condition effect	two-phased intervention (FECs + public posting of aggregate MDT scores = secondary M7); mixed sample, 80.6% nurses (population adjudication: team- based, precedent Sporrong/van der Dam); identified via citation searching (July 2026)

Abbreviations: CD-RISC, Connor-Davidson Resilience Scale; CLCDI, Comfort Level Communication with Dying Infants; FEC, facilitated ethics conversation; GES, Global Empowerment Scale; MBSR, mindfulness-based stress reduction; MD, moral distress; MDS, Moral Distress Scale; MDS-R, Moral Distress Scale-Revised; MDT, Moral Distress Thermometer; MMD-HP, Measure of Moral Distress for Healthcare Professionals; QWC, Quality Work Competence.

Supplementary File S5. Sensitivity analysis of the evidence gap map under multiple coding

The evidence gap map (Figure 2 of the main text) assigns each study to a single cell defined by its primary modality, its intervention level and its primary point of action. This file reports the same distribution under multiple coding, in which each study is counted in every modality and every point of action for which it holds either a primary or a secondary code in Supplementary File S4, Panel B, and in every combination of those codes in the cross-tabulation. Sixteen of the 32 studies carry at least one secondary code; under multiple coding the 32 studies contribute 70 study-cell entries to the modality-by-point-of-action table. Intervention level is coded once per study and is therefore unaffected. The analysis was computed directly from the charted dataset with the coding strategy as the only variable.

Table S5.1. Marginal frequencies by dimension under primary and multiple coding (n = 32 studies)

Dimension and category	Primary coding	Multiple coding	Studies contributing a secondary code
M1 Education	13	13	none
M2 Facilitated case discussion and debriefing	2	7	Brandon [26]; Bruce & Allen [29]; Davis & Batcheller [31]; Meziane [39]; Vaclavik [48]
M3 Ethics deliberation and consultation services	9	13	Davis & Batcheller [31]; Meziane [39]; Kälvemark Sporrong [52]; Wocial 2017 [50]
M4 Interprofessional rounds	1	2	Brandon
M5 Reflective and narrative methods	3	4	Bevan & Emerson [24]
M6 Resilience and mindfulness methods	2	4	Beumer [23]; Rushton [45]
M7 Structural-organisational measures	2	4	Tavakol [47]; Wocial 2024 [51]
A1 Preventive, at the source	1	4	Epstein 2023 [32]; Hamric & Epstein [32]; Tavakol [47]
A2 Supportive-enabling, around the moral event	24	28	Browning & Cruz [28]; Davis & Batcheller [31]; Jakobsen [36]; Meziane [39]
A3 Processing-downstream	7	12	Beumer [23]; Brandon [26]; Bruce & Allen [29]; Rushton [45]; Wocial 2024 [51]
L1 Individual	11	11	not applicable (single code)
L2 Team or unit	16	16	not applicable (single code)
L3 Organisation or system	5	5	not applicable (single code)

Under multiple coding, the marginal counts by modality sum to 47 and the counts by point of action to 44, because a study with a secondary code is counted twice; under primary coding both sum to 32.

Table S5.2. Modality against point of action under primary and multiple coding (cell entries: primary / multiple)

Modality	A1 at the source	A2 around the event	A3 downstream	Row total

Modality	A1 at the source	A2 around the event	A3 downstream	Row total
M1 Education	0 / 1	13 / 13	0 / 3	13 / 17
M2 Facilitated case discussion and debriefing	0 / 0	0 / 5	2 / 7	2 / 12
M3 Ethics deliberation and consultation services	0 / 2	9 / 13	0 / 4	9 / 19
M4 Interprofessional rounds	0 / 0	1 / 2	0 / 1	1 / 3
M5 Reflective and narrative methods	0 / 0	0 / 3	3 / 3	3 / 6
M6 Resilience and mindfulness methods	0 / 0	0 / 3	2 / 4	2 / 7
M7 Structural-organisational measures	1 / 2	1 / 3	0 / 1	2 / 6
Column total	1 / 5	24 / 42	7 / 23	32 / 70

Under multiple coding every combination of a study's modality codes with its point-of-action codes is counted, so that a study with two modality codes and two point-of-action codes contributes four entries; the column total for A1 (5) therefore exceeds the number of studies holding an A1 code (4), because Tavakol holds both M1 and M7.

Table S5.3. Modality against point of action under multiple coding, stratified by intervention level

Modality	L1: A1 / A2 / A3	L2: A1 / A2 / A3	L3: A1 / A2 / A3
M1	0 / 9 / 2	1 / 4 / 1	0 / 0 / 0
M2	0 / 0 / 0	0 / 4 / 6	0 / 1 / 1
M3	0 / 0 / 0	0 / 10 / 3	2 / 3 / 1
M4	0 / 0 / 0	0 / 1 / 0	0 / 1 / 1
M5	0 / 1 / 2	0 / 2 / 1	0 / 0 / 0
M6	0 / 2 / 2	0 / 1 / 2	0 / 0 / 0
M7	0 / 0 / 0	1 / 2 / 1	1 / 1 / 0

Interpretation. The principal pattern of the map is robust to the coding strategy. Action at the source remains the least populated region under multiple coding (4 of 32 studies, three of them as a secondary aim), the organisational or system level is unaffected (5 of 32), and education remains the most frequent modality, now equalled by ethics deliberation and consultation (13 each). The only substantive shifts are the increase in processing-downstream action from 7 to 12 entries, reflecting the debriefing and resilience components embedded in several

integrated programmes, and the increase in M2 and M3 as secondary components. Multiple coding accordingly renders the map fuller without altering the location of its gaps.

Supplementary File S6. Reported direction of effect by study design and by the fourth MMAT criterion

The fourth criterion of the Mixed Methods Appraisal Tool (MMAT) 2018 is design-specific and does not assess the same threat to validity across categories (Table S6.1). It assesses the control of confounding only in the non-randomised category (item 3.4). For this reason the reported direction of effect on moral distress, charted in Supplementary File S4, Panel B, is tabulated here against study design (Table S6.2), and the aggregate statement in Section 3.7 of the main text is decomposed by category. Study design follows the charting in Supplementary File S4, Panel A. Three strata are distinguished: randomised trials; non-randomised studies with any comparison condition (a concurrent non-equivalent group, a historical control group, a cross-over comparison, or a non-equivalent group surveyed before the intervention); and uncontrolled designs (one-group pre/post, within-subject repeated measures, and repeated or separate-sample cross-sectional surveys without a comparison group). The 11 studies that did not permit a usable inference about the direction of effect on a direct moral-distress measure are listed separately.

Table S6.1. What the fourth MMAT criterion assesses in each design category, with its ratings in the corpus

MMAT category	n	Fourth criterion (MMAT 2018 item)	Yes	Can't tell	No
1 Qualitative	3	Is the interpretation of results sufficiently substantiated by data? (1.4)	1	2	0
2 Quantitative randomised controlled trials	4	Are outcome assessors blinded to the intervention provided? (2.4)	0	3	1
3 Quantitative non-randomised	13	Are the confounders accounted for in the design and analysis? (3.4)	1	6	6
4 Quantitative descriptive	2	Is the risk of nonresponse bias low? (4.4)	0	2	0
5 Mixed methods	10	Are divergences and inconsistencies between quantitative and qualitative results adequately addressed? (5.4)	0	10	0
All categories	32		2	23	7

Table S6.2. Reported direction of effect on a direct moral-distress measure by design stratum (21 evaluable studies)

Design stratum	Study (reference)	MMAT category and design as charted	Reported direction	Fourth criterion

Design stratum	Study (reference)	MMAT category and design as charted	Reported direction	Fourth criterion
**Randomised trials (n = 4)**	Abbasi et al. 2019 [21]	RCT, two-arm parallel; control arm received a brief intervention	Reduction	Can't tell (2.4)
	Molazem et al. 2013 [40]	RCT, two-arm parallel, permuted blocks	Reduction	Can't tell (2.4)
	Saeedi et al. 2019 [46]	RCT, single-blind, two-arm parallel	No change	Can't tell (2.4)
	Brännström et al. 2025 [27]	Cluster RCT, open	No change	No (2.4)
**Non-randomised with a comparison condition (n = 5)**	Epstein et al. 2023 [32]	Mixed methods; pre/post in consultation participants with a non-equivalent control group of unit staff surveyed before the consultation	Reduction	Can't tell (5.4)
	Browning & Cruz 2018 [28]	Non-randomised, non-equivalent control group, pre/post	No change	Can't tell (3.4)
	Rushton et al. 2021 [45]	Non-randomised, comparison group surveyed once, ANCOVA	No change	Yes (3.4)
	Wocial et al. 2017 [50]	Non-randomised, pre/post with historical controls	No change	Can't tell (3.4)
	Wocial et al. 2024 [51]	Mixed methods; cross-over quasi-experimental comparison between conditions	No change	Can't tell (5.4)
**Uncontrolled designs (n = 12)**	Chiafery et al. 2018 [30]	Mixed methods; one-group pre/post	Reduction	Can't tell (5.4)
	Monteverde 2016 [41]	Non-randomised; within-subject repeated measures	Reduction	Can't tell (3.4)
	Robinson et al. 2014 [43]	Mixed methods; one-group pre/post	Reduction	Can't tell (5.4)
	Tavakol et al. 2023 [47]	Mixed methods; one-group pre/post	Reduction	Can't tell (5.4)
	Vaclavik et al. 2018 [48]	Non-randomised; one-group pre/post	Reduction	No (3.4)
	Bevan & Emerson 2020	Mixed methods; one-group pre/post	Mixed	Can't tell (5.4)

Design stratum	Study (reference)	MMAT category and design as charted	Reported direction	Fourth criterion
	[24]			
	Bosshardt et al. 2018 [25]	Mixed methods; repeated cross-sectional survey	Mixed	Can't tell (5.4)
	Brandon et al. 2014 [26]	Non-randomised; unpaired pre/post	Mixed	Can't tell (3.4)
	Meziane et al. 2018 [39]	Mixed methods; pre/post	Mixed	Can't tell (5.4)
	Bruce & Allen 2020 [29]	Non-randomised; one-group pre/post	No change	No (3.4)
	Fontenot & White 2019 [33]	Non-randomised; one-group pre/post	No change	No (3.4)
	Leggett et al. 2013 [37]	Non-randomised; separate-sample pre/post	Increase	Can't tell (3.4)
**Not evaluable on a direct moral-distress measure (n = 11)**	Allen & Butler 2016 [22]; Beumer 2008 [23]; Davis & Batcheller 2020 [31]; Hamric & Epstein 2017 [34]; Iaboni et al. 2022 [35]; Jakobsen et al. 2023 [36]; Lillemoen & Pedersen 2015 [38]; Reilly & Jurchak 2017 [42]; Rogers et al. 2008 [44]; Kälvemark Sporrong et al. 2007 [52]; van der Dam et al. 2011 [49]	Descriptive, qualitative, surrogate-outcome or non-validated-instrument studies (see Supplementary File S4, Panel B)	No usable inference	Yes 1; can't tell 7; no 3

Table S6.3. Summary of reported direction by design stratum

Design stratum	n	Reduction	Mixed	No change	Increase
Randomised trials	4	2	0	2	0
Non-randomised with a comparison condition	5	1	0	4	0
Uncontrolled designs	12	5	4	2	1

Design stratum	n	Reduction	Mixed	No change	Increase
All evaluable studies	21	8	4	8	1

Interpretation. Read by design, the reductions are not confined to weak designs: two of the four randomised trials reported a reduction, although in neither could the blinding of outcome assessment be established from the report. Five of the eight reductions derive from uncontrolled designs and a sixth from a non-equivalent comparison in which the reduction was observed as a within-group change among consultation participants. Within the non-randomised category, the only stratum in which the fourth criterion assesses confounding, both studies reporting a reduction were rated “no” or “can’t tell” on that item, but so were five of the six non-randomised studies reporting no change or a mixed pattern, so that the criterion does not discriminate between directions in this corpus. The statement in the main text has accordingly been confined to the description that most reported reductions derive from uncontrolled designs; no inference about effectiveness is drawn.
